# Correcting PCR amplification errors in unique molecular identifiers to generate accurate numbers of sequencing molecules

**DOI:** 10.1038/s41592-024-02168-y

**Published:** 2024-02-05

**Authors:** Jianfeng Sun, Martin Philpott, Danson Loi, Shuang Li, Pablo Monteagudo-Mesas, Gabriela Hoffman, Jonathan Robson, Neelam Mehta, Vicki Gamble, Tom Brown, Tom Brown, Stefan Canzar, Udo Oppermann, Adam P. Cribbs

**Affiliations:** 1 Botnar Research Centre, Nuffield Department of Orthopaedics, Rheumatology and Musculoskeletal Sciences, National Institute of Health Research Oxford Biomedical Research Unit (BRU), University of Oxford, Oxford, UK; 2 Gene Center, Ludwig-Maximilians University of Munich, Munich, Germany; 3 ATDBio Ltd (now part of Biotage), Magdalen Centre, Oxford Science Park, Oxford, UK; 4 Chemistry Research Laboratory, Department of Chemistry, University of Oxford, Oxford, UK; 5 Department of Computer Science and Engineering, The Pennsylvania State University, University Park, PA USA; 6 Huck Institutes of the Life Sciences, The Pennsylvania State University, University Park, PA USA; 7 Oxford Centre for Translational Myeloma Research, University of Oxford, Oxford, UK

**Keywords:** RNA sequencing, Transcriptomics, Computational platforms and environments

## Abstract

Unique molecular identifiers are random oligonucleotide sequences that remove PCR amplification biases. However, the impact that PCR associated sequencing errors have on the accuracy of generating absolute counts of RNA molecules is underappreciated. We show that PCR errors are a source of inaccuracy in both bulk and single-cell sequencing data, and synthesizing unique molecular identifiers using homotrimeric nucleotide blocks provides an error-correcting solution that allows absolute counting of sequenced molecules.

## Abstract (web summary)

This study introduces a method utilizing homotrimeric nucleotide blocks to achieve accurate counts of RNA molecules in both bulk and single-cell sequencing data.

## Main

Unique molecular identifiers (UMIs)^1^ distinguish molecules in sequencing, enabling correction for biases in sampling and PCR amplification across next-generation and third-generation sequencing methods, including bulk RNA^2,3^, single-cell RNA^4,5^ and DNA approaches^6–8^. However, the accuracy of molecular quantification can be affected by the varying sequencing quality of different platforms^9^. Different sequencing platforms necessitate varied PCR cycling conditions, potentially introducing UMI errors that may result in inaccurate molecule counts (Supplementary Fig. 1). Unlike sample barcodes for multiplexing or cell barcodes in single-cell sequencing, which can be whitelisted due to a limited pool of barcodes^10^, UMIs cannot be corrected using this approach as their synthesis is random. Therefore, UMIs are often corrected using computational approaches^11^, concatemeric consensus sequencing^12^ or by bespoke UMI designs^13,14^. Despite several computational approaches that leverage Hamming distances^15,16^, graph networks^11,13^ or thresholding on UMI frequency^4^, experimental validation of these solutions is lacking, with simulations indicating persistent UMI errors postcomputational demultiplexing^13^.

**Fig. 1:**
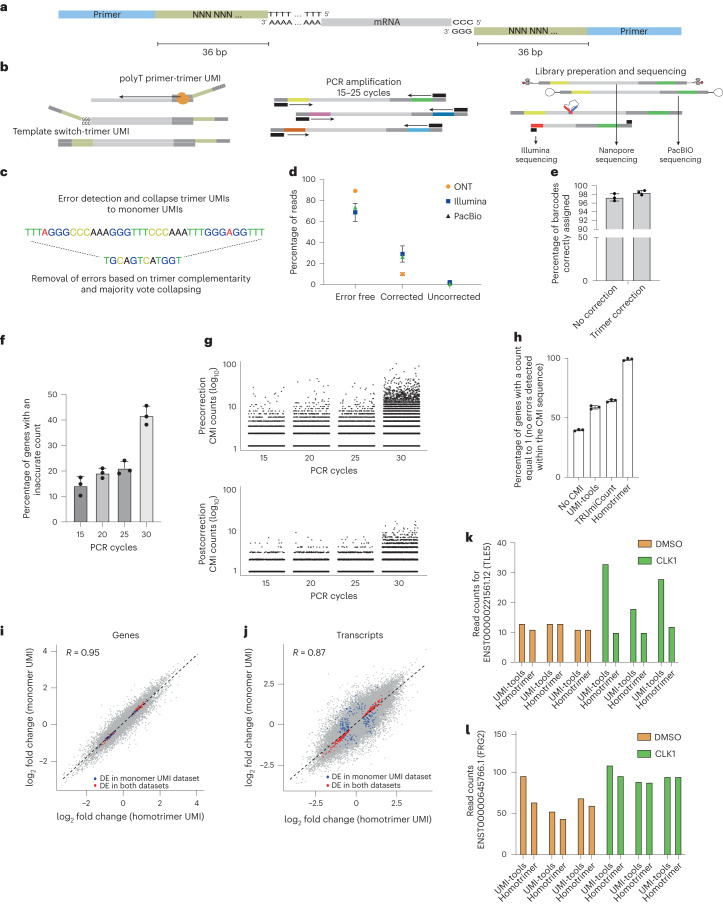
Enhanced accuracy in bulk mRNA sequencing using homotrimer UMI-based approach to mitigate PCR-induced errors. **a**, A schematic showing attachment of 3′ and 5′ UMIs to mRNA. **b**, A schematic showing the homotrimeric UMI approach. **c**, Errors are then corrected using the homotrimer correction method. **d**, Percentage of CMIs that are correctly sequenced and then error corrected using homotrimer correction across Illumina, PacBio and ONT sequencing platforms. Experiments for Illumina and ONT were performed in triplicate, whereas PacBio sequencing was conducted as a single run. Parameters for simulations: sequencing error rate 0.001, length of UMI 8, PCR cycles 10 and PCR error rate 0.000001 **e**, Barcode assignment using homotrimer barcodes before and after majority vote correction. **f**, Percentage of genes with an accurate CMI count following increased PCR cycles of the same sequencing library. Data shown in the figure are from one single run. **g**, log_10_ CMI counts plotted for each transcript pre- and postmajority vote correction. Each dot represents an individual transcript (the ground truth count for each transcript should be equal to 1, any counts above this are indicative of an error). The data in this figure are representative of one sample in **f**. **h**, Percentage of genes with an accurate CMI count following 20 PCR cycles then using ONT sequencing and counting using UMI-tools, TRUmiCount correction and homotrimer error correction. **i**–**l**, RM82 sarcoma cells were treated with DMSO or SGC-CLK-1 for 24 hours and then sequenced using the PromethION platform. **i**,**j**, Scatter plot of the log_2_ fold changes obtained from randomly collapsing each sequenced trimer UMI and then applying UMI-tools deduplication versus the log_2_ fold changes obtained from homotrimer UMI correction and counting for genes (**i**) and transcripts (**j**). Red points indicate the overlapping significant genes and/or transcripts and blue points indicate genes and/or transcripts that were disconcordantly significantly differentially expressed. DE, differential expression. **k**, TLE5 transcript read counts showing the expression for DMSO and SGC-CLK1 following the application of UMI-tools or homotrimer correction. **l**, FRG2 transcript read counts showing the expression for DMSO and SGC-CLK1 following the application of UMI-tools or homotrimer correction. For **k** and **l**, three replicates are shown for each condition. **d**,**e**,**f** and **h**, Error bars represent standard deviation (s.d.) from three independent experiments.

We reasoned that using homotrimer nucleotides to synthesize UMIs would simplify error detection and correction by using a ‘majority vote’ method (Fig. 1a and Supplementary Fig. 2). Our method labels RNA with homotrimeric UMIs at either end for enhanced error detection and indel tolerance, compatible with the ONT (Oxford Nanopore Technologies), PacBio or Illumina platforms (Fig. 1a,b). UMIs are processed by assessing trimer nucleotide similarity; errors are corrected by adopting the most frequent nucleotide in a majority vote approach (Fig. 1c). Our simulations reveal that a demultiplexing strategy incorporating homotrimers, along with a set coverage approach, outperforms the existing gold standard of monomer-based UMI-tools demultiplexing (Supplementary Fig. 3). By synergistically integrating homotrimeric correction and set coverage techniques, our method achieves a substantial improvement in the detection and recovery of simulated UMIs. This optimized performance exceeds the results obtained when relying solely on a homotrimer majority vote approach (Supplementary Fig. 4).

While sequencing simulations can offer valuable insights, their real-world applicability may be limited by biases. To validate our homotrimer UMI error correction approach, we conducted experiments using a common molecular identifier (CMI) attached to every captured RNA molecule (Supplementary Fig. 5). Having the same molecule attached to every RNA guarantees that, in the absence of errors, each transcript is only counted once. However, if errors are introduced into the CMI, transcripts will be overcounted. This provides a means for assessing the accuracy of library preparation and sequencing, as well as the impact of errors on the transcript counts (Supplementary Fig. 5b).

We attached the CMI to equimolar concentrations of mouse and human complementary DNA (cDNA) at the 3′ end, PCR amplified and then split the sample for sequencing on Illumina, PacBio or ONT platforms. We calculated the Hamming distance between the observed and expected CMI sequence to measure sequencing accuracy. Our results show that 73.36, 68.08 and 89.95% of CMIs were correctly called using Illumina, PacBio and the latest kit14 ONT chemistry, respectively (Fig. 1d and Supplementary Figs. 6–8). Older ONT chemistry gave substantially lower accuracy (Supplementary Fig. 9), but the use of super accuracy base calling led to substantial improvements (Supplementary Fig. 10). Using our homotrimeric error correction approach, we were able to correctly call 98.45, 99.64 and 99.03% of CMIs for Illumina, PacBio and the latest ONT chemistry, respectively (Fig. 1d). We hypothesized that the lower accuracy of Illumina and PacBio, when compared to ONT sequencing, may be due to the use of polymerases that are integral to the sequencing process (for example, bridge amplification^17^ and circular consensus sequencing^18^ with Illumina and PacBio, respectively). To discern sequencing and PCR errors, we amplified a CMI-tagged cDNA library with increasing PCR cycles and sequenced using ONT’s Minion. Trimer barcodes added during PCR allowed for batch effect minimization and independent sequencing accuracy assessment. High barcode accuracy was noted, with homotrimer correction offering negligible improvement (Fig. 1e). Based on these results, it can be inferred that sequencing errors make a negligible contribution to the overall error rate. However, we observed a substantial increase in the number of errors within our CMIs with increasing PCR cycles (Fig. 1f and Supplementary Fig. 11). Our homotrimer approach was able to correct a significant proportion of errors observed within the CMIs (Fig. 1g). This suggests that PCR can be a significant source of UMI error. We also benchmarked homotrimer error correction against both UMI-tools^11^ and TRUmiCount^19^ and found substantial improvements in error correction (Fig. 1h and Supplementary Fig. 12). We observed minimal indel errors, suggesting that most errors were substitutions (Supplementary Fig. 12). It is important to highlight that monomer UMIs using Hamming distance, such as those in UMI-tools and TRUmiCount, cannot correct indel errors due to the potential for a single indel to inflate the Hamming distance beyond correctability. Our methodology overcomes this by including indel correction.

We next conducted an experiment to correct PCR errors using homotrimers and treated Ewing’s RM82 sarcoma cells with a CLK1 splicing kinase inhibitor. This induced splicing perturbations, allowing observation of an exaggerated differential transcript effect, followed by ONT or Illumina sequencing (Fig. 1i–l and Supplementary Figs. 13 and 14). When we compared monomer UMI correction to our homotrimer correction methodology, we found differences in the number of differentially expressed genes and transcripts between splicing inhibition and control conditions. Specifically, for genes and transcripts, we observed discordant rates of 7.8 and 11%, respectively (Fig. 1i–j and Supplementary Tables 2 and 3). The discordance rate indicates exclusive gene or transcript regulation in one condition over another. More genes were differentially regulated after monomer UMI-tools correction than homotrimer correction, exemplified by read counts for TEL5 and FRG2 genes postcorrection (Fig. 1k and Supplementary Fig. 15). In addition, the homotrimer correction approach led to an increased fold enrichment of genes associated with gene ontology terms related to DNA replication and splicing (Supplementary Fig. 16), highlighting the improved accuracy of our method in identifying biologically relevant gene sets. Additionally, we also observed 4.7% discordant differentially expressed genes between UMI-tools and homotrimer correction following Illumina sequencing (Supplementary Fig. 13c,d).

**Fig. 2:**
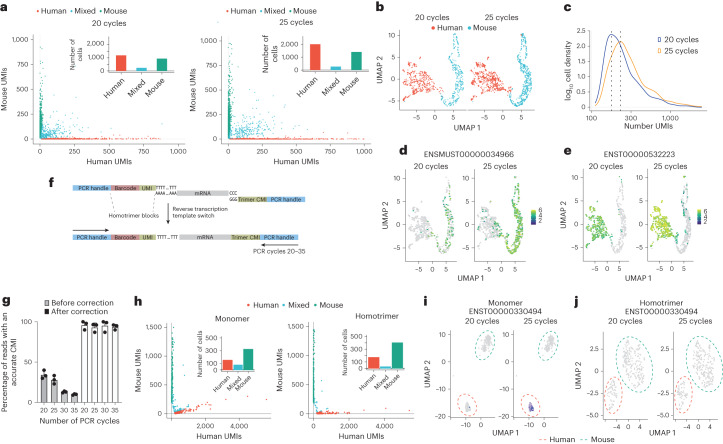
Homotrimer UMIs enhance differential expression accuracy in single-cell sequencing. **a**, Human Jurkat and mouse 5TGM1 cells were mixed for encapsulation and cDNA synthesis using 10X chromium followed by nanopore sequencing. Each dot represents an individual cell. **b**, A UMAP of 10X chromium data showing the integration, clustering and annotation of human and mouse cells following 20 and 25 cycles of PCR. **c**, A density plot for the 10X chromium data showing the log_10_ density of the number of UMIs following 20 and 25 cycles of PCR. The dotted line shows the maximum density for each condition. **d**, A UMAP showing the expression of ENSMUST00000034966 between libraries amplified following 20 and 25 PCR cycles. **e**, A UMAP showing the expression of ENST00000532223 between libraries amplified following 20 and 25 cycles of PCR. **f**, A schematic showing the homotrimer UMI drop-seq library preparation approach and template switching attachment of a homotrimer CMI to single-cell captured mRNAs. **g**, Drop-seq libraries were sequenced using the Flongle sequencing device, graphs show the percentage of reads that have an accurate CMI following amplification of the same library using 20, 25, 30 and 35 cycles of PCR before and after homotrimer correction. Error bars are the s.d. of three independent experiments. **h**, Barnyard plots showing the expression of mouse and human cells following 20 and 25 cycles of PCR and sequencing using a PromethION sequencing device. Each dot represents an individual cell. **i**,**j**, UMAP plots showing the transcript expression of ENST00000330494 following monomer-based UMI-tools demultiplexing (**i**) and homotrimer-based demultiplexing (**j**). In each UMAP plot, each dot represents a cell. Data shown in **g** were collected in a single sequencing run and *n* = 1.

To understand the effect of PCR errors on single-cell sequencing accuracy, we used the 10X Chromium system with monomer UMIs to encapsulate JJN3 human and 5TGM1 mouse cells, followed by ten PCR cycles. Subsequently, we divided the PCR product into two portions and performed additional PCR amplification, resulting in a combined number of PCR cycles of 20 or 25. We then prepared and sequenced these libraries using ONT’s PromethION platform and after assigning cell barcodes (Fig. 2a) and filtering, clustering and annotating the cells (Fig. 2b), we observed that the library subjected to 25 cycles of PCR had a greater number of UMIs compared to the library that underwent 20 PCR cycles (Fig. 2c and Supplementary Fig. 17). This suggests that PCR errors contribute to inaccurate counting of transcripts and an inflated UMI count. We next performed differential gene expression and identified 50 differentially expressed transcripts (Supplementary Tables 4 and 5). For example, transcripts ENSMUST00000034966 (Fig. 2d; Rpl4, ribosomal protein L4) and ENST00000532223 (Fig. 2e; IGLL5, immunoglobulin lambda), were identified as significant (*P*_adj_ < 0.05) in this differential expression analysis, highlighting the contribution of PCR errors to inaccurate transcript counting.

Next, we encapsulated JJN3 human and 5TGM1 mouse cells using Drop-seq^20^ with trimer barcoded beads, conducted reverse transcription and template switching with a CMI and initiated ten PCR cycles. The PCR product was split into four aliquots for further amplification to 20, 25, 30 and 35 PCR cycles, respectively, before sequencing on the ONT Minion platform. Our results indicate a decrease in the percentage of reads with accurate CMIs as the number of PCR cycles increases. We show that homotrimer correction leads to 96–100% correction of CMI sequences (Fig. 2g and Supplementary Fig 18). This underscores the effectiveness of this approach in removing errors introduced by PCR. Subsequently, we sequenced the libraries that underwent 20 or 25 PCR cycles using ONT’s PromethION platform (Supplementary Fig. 19). Our results show that by incorporating homotrimers within the barcode region an increase, albeit low (~15%), in the numbers of cells recovered was achieved (Fig. 2h). Monomeric UMI deduplication resulted in over 300 differentially regulated transcripts between the 20 and 25 cycle libraries (Fig. 2i and Supplementary Table 4). On the contrary, the application of homotrimer correction did not reveal any significantly differentially regulated transcripts. Transcripts with high counts after 25 cycles of monomer UMI correction saw a reduction when subjected to homotrimer UMI correction (Fig. 2j), demonstrating the robustness of homotrimer UMIs to remove errors.

Our research highlights the importance of accurate UMI quantification in sequencing, endorsing homotrimer UMIs to improve read count precision. Homotrimers notably mitigate PCR-induced UMI errors, optimizing molecule count sequencing. Although they extend the oligonucleotide length, their suitability for long-read sequencing—unrestricted by read length—outweighs this limitation, offering substantial benefits for studies requiring rigorous UMI quantification.

## Methods

### Cell lines and reagents

The 5TGM1, Jurkat and RM82 cell lines were cultured in complete Roswell Park Memorial Institute medium. All parental cell lines were tested twice per year for mycoplasma contamination and authenticated by short tandem repeat during this project. For cell culture experiments, SGC-CLK-1 (Structural Genomics Consortium) inhibitor was incubated with cells for 24 h. Dimethylsulfoxide (DMSO) was used as a negative control. Jurkat (TIB-152) were purchased from the American Type Culture Collection, while 5TGM1 cells were a kind gift from C. Edwards (University of Oxford) and RM82 cells were gifts from N. Athanasou and B. Hassan, as part of the EuroBioNet network.

### Oligonucleotide synthesis

Homotrimer phosphoramidites were purchased as a custom product from Metkinen Chemistry and reverse homotrimer phosphoramidites were a custom synthesis product from Chemgenes. Solid-phase phosphoramidite oligonucleotide synthesis on Toyopearl HW-65S resin (Tosoh Biosciences, catalog no. 0019815) was performed by ATDBio as described previously^13^, in the 5′–3′ direction (using reverse amidites), using a method adapted from ref. ^20^. The sequence of the capture oligonucleotide is as follows: Bead-5′-[spacer]-TTTTTTTAAGCAGTGGTATCAACGCAGAGTACJJJJJJJJJJJJNNNNNNNNTTTTTTTTTTTTTTTTTTTTTTTTTTTTTT-3′, where ‘J’ indicates a nucleotide trimer block added via split-and-pool synthesis using reverse monomer phosphoramidites. ‘N’ indicates a degenerate trimer nucleotide (added using an equimolar mixture of the four reverse timer phosphoramidites). [spacer] is hexaethylene glycol added using DMT-protected hexaethylene glycol phosphoramidite and all the other bases are standard (monomeric) DNA bases added using reverse phosphoramidites. AAGCAGTGGTATCAACGCAGAGTAC is the PCR handle.

Before oligonucleotide synthesis, capping was performed to reduce the initial loading of hydroxyl groups on the beads, by suspending the resin in a 1:1 mixture of Cap A (tetrahydrofuran:lutidine:acetic anhydride 8:1:1) and Cap B (tetrahydrofuran:pyridine:1-methylimidazole 8:1:1) at room temperature for 30 min. Oligonucleotide synthesis was then performed using an ABI 394 DNA synthesizer, using a modified 1 μmol synthesis cycle (with an extended coupling time of 5 min for monomer bases and 10 min for trimer bases. The capping step was omitted for the trimer bases in the UMI region and the poly-T region). The barcode was generated using 12 split-and-pool synthesis cycles. Before the first split-and-pool synthesis cycle, beads were removed from the synthesis column, pooled and mixed, and divided into four equal aliquots. The bead aliquots were then transferred to separate synthesis columns before three consecutive couplings with monomers reverse amidites. This process was repeated 11 times. Following the final split-and-pool cycle, the beads were pooled, mixed and divided between four columns, ready for the next part of the synthesis. An equimolar mixture of the four trimer phosphoramidites was used in the synthesis of the degenerate UMI (poly(N)) region, and (monomeric) T reverse amidite was used for the poly(T) tail. After oligonucleotide synthesis, the resin was washed with acetonitrile and dried with argon before deprotection in aqueous ammonia (room temperature for 17 h followed by 55 °C, 6 h). The beads were then washed with water followed by acetonitrile and dried with argon gas.

Template switch oligonucleotide was synthesized using standard phosphoramidites: 5′-AAGCAGTGGTATCAACGCAGAGTNNNNNNNNNNGAATrGrGrG-3′. The oligonucleotides were PAGE purified and shipped lyophilized. Primers containing CMIs were synthesized by Sigma Aldrich using the following sequences polyA oligonucleotide: 5′-AAGCAGTGGTATCAACGCAGAGTACNNNNNNNNNNTTTTTTTTTTTTTTTTTTTTTTTTTTTTTT-3′.

### Generating bulk homotrimer UMI-tagged cDNA

Total mesenger RNA (mRNA) was isolated using a Quick-RNA MiniPrep kit (Zymo), following the manufacturer’s protocol. The RNA sample quality and quantity was measured using an RNA screen tape on the TapeStation (Agilent). cDNA synthesis was performed with modification to the SMART approach^21^. An oligo(dT)-containing adapter containing a homotrimer 30-base DNA sequence and a SMART primer sequence was used to initiate a reverse transcriptase reaction. Briefly, RNA was denatured at 72 °C for 2 min and then reverse transcribed with Maxima H minus reverse transcriptase (2,000 U) in a total volume of 50 μl with the buffer, 1 mM dNTPs, 2 mM dithiothreitol and 4% Ficoll PM-400. The reaction was performed for 90 min at 42 °C and then the enzyme was heat inactivated at 80 °C for 5 min. The library was then purified using 0.8× SPRI bead (Beckman Coulter) cleanup followed by PCR using KAPA HiFi master mix for 20 cycles (unless otherwise stated)^20^, using SMART PCR primer (AAGCAGTGGTATCAACGCAGAGT) before being purified using SPRI beads. To achieve a high concentration of cDNA the input was subjected to up to 30 cycles of PCR amplification followed by a second cleanup. Optionally, 10 ng of PCR product was subjected to 12 further cycles of PCR using primers that contained trimer sample barcodes (Supplementary Table 1). Finally, cDNA was quantified using a TapeStation (Agilent Technologies) using a DNA high-sensitivity D5000 tape before being split for Illumina or Oxford Nanopore library generation. To reduce PCR artifacts and improve sequencing return, we performed PCR using the primer 5-PCBio-TACACGACGCTCTTCCGATCT for a further 3–5 cycles of PCR.

### ONT bulk RNA sequencing (RNA-seq) library preparation and sequencing

A total of 1,200 ng of purified cDNA was used as a template for ONT library preparation. We used SQK-LSK-109, SQK-LSK112 and SQK-LSK114 (also referred to as ONT latest kit14 chemistry) ligation sequencing kits, following the manufacturer’s protocol. Samples were sequenced using a minION device using R9.4.1 (FLO-MIN106D) or R10.4 (FLO-MIN112) flow cells. Barcoding using the Native Barcoding Amplicon kit (EXP-NDB104) was performed for RM82 cells treated with DMSO or CLK1 inhibitor treatment. These samples were sequenced using the PromethION sequencing platform on R9.4.1 FLO-PRO002 flow cells at the Deep Seq facility at the University of Nottingham.

### PacBio bulk RNA-seq library preparation and sequencing

A total of 1,200 ng of purified cDNA was used as a template for PacBio library preparation and sequencing at the Centre for Genomic Research at the University of Liverpool (https://www.liverpool.ac.uk/genomic-research/technologies/next-generation-sequencing/). cDNA was end-repair and A-tailed with T4 polynucleotide kinase (New England Biolabs). The sequencing library was prepared using the SMRTbell Express Template Prep Kit v.2.0 following the standard protocol. Sequencing was then performed on a Sequel IIe using a Sequel IIe SMRT Cell 8M ion CCS mode, following the standard protocol. CCS reads were generated using CCS v.6.3.0 (https://github.com/PacificBiosciences/ccs) using default settings.

### Illumina bulk RNA-seq library preparation and sequencing

Purified cDNA was used as an input for the Nextera XT DNA library preparation kit (New England Biolabs). Library quality and size was determined using a TapeStation (Agilent Technologies) High Sensitivity D1000 tape and then sequenced on a NextSeq 500 sequencer (Illumina) using a 75-cycle High Output kit using a custom read1 primer (GCCTGTCCGCGGAAGCAGTGGTATCAACGCAGAGTAC). Read1 length was 30 bp long and read2 length was 52 bp long.

### ONT bulk RNA-seq analysis

We performed base calling on the raw fast5 data to generate fastq files using Guppy (v.6.4.8) (guppy_basecaller –compress-fastq -c [cfg file] -x ‘cuda:0’) in graphical processing unit (GPU) mode from ONT running on a RTX3090 graphics card. The fastq data were processed using a custom pipeline ‘pipeline_count’ written using cgatcore^22^ and included within the TallyTriN repository. Briefly, the quality of each fastq file was evaluated using the fastqc toolkit^23^ and summary statistics were collated using Multiqc^24^. We then identified the polyA associated UMI sequence by searching for the polyA region and reverse complementing the read if it did not appear in the correct orientation. The 30 bp UMI was identified upstream of the SMART primer by pattern matching for GTACTCTGCGTTGATACCACTGCTT. The set coverage method for removing homotrimer methods was then applied for UMI demultiplexing; if the UMI contained more than five errors then the read was removed. The demultiplexed UMI sequence was then added to the read name. Next, the template switch oligo (TSO)-associated UMI was identified using the SMART primer sequence AAGCAGTGGTATCAACGCAGAGTAAT. The 30 bp UMI sequence was first subjected to error correction or removed from the read, depending on the number of UMI errors detected. Subsequently, the corrected UMI, if applicable, was appended to the read name, enhancing the accuracy and use of the data. Both the TSO and polyA associated UMIs and primer sequences were removed from the read sequence. For transcript level analysis, the fastq file was then mapped against the transcriptome using minimap2 (v.2.25) with the following settings: -ax map-ont -p 0.9 --end-bonus 10 -N 3. The resulting .sam file was then sorted and indexed using samtools^25^. A custom script, in which pysam (v.0.21.0) was used to parse the output .sam file, was then used to add the transcript name to the XT tag of the samfile for downstream counting by homotrimer deduplication or UMI-tools. For gene level analysis, the fastq data were mapped using minimap2 using the following setting: -ax splice -k 14 --sam-hit-only --secondary=no --junc-bed. The resulting .sam file was then sorted and indexed followed by feature annotation using featurecounts (v.2.0.1)^26^ using the following settings to generate an annotated .bam file: featureCounts -a (gtf) -o (output) -R BAM. This .bam file was then used for downstream counting by UMI-tools or homotrimer correction. The reference transcriptome and genomes used for the analysis were hg38_ensembl98 and mm10_ensembl88. The resulting count tables were then used for differential gene expression analysis, which was performed using DESeq2 v.1.40.2 (ref. ^27^) within the R statistical framework v.4.3.1.

### Illumina bulk RNA-seq analysis

The data were processed using a custom cgatcore^28^ (v.0.6.15) written pipeline ‘pipeline_illumina’. Briefly, the UMIs contained in read1 were corrected based on homotrimer complementarity or were removed from the analysis depending on a set error threshold. The paired fastq files were then mapped using hisat2 (v.2.2.1)^29^ before features being counted using featureCounts^30^ (v.2.0.3) using the following commands: featureCounts -a (gtf) -o (output) -R BAM. The resulting XT tagged .bam file was then used for downstream counting using homotrimer deduplication or UMI-tools. The resulting count tables were then used for differential gene expression analysis, which was performed using DESeq2 v.1.40.2 (ref. ^27^) within the R statistical framework v.4.3.1

### UMI-tools deduplication

Following gene or transcript level mapping, the UMI was extracted from the read. Since UMI-tools was not designed to correct homotrimer sequences, we collapsed the UMI into a single nucleotide sequence by selecting the first base within each of the individual trimers. Reads were then deduplicated using the directional method using the command: umi_tools count –per-gene –gene-tag=XT.

### Homotrimer set coverage deduplication

Following gene or transcript level mapping, the UMI was extracted from the read and collapsed into single nucleotide sequence using the majority vote approach where applicable or resolve inconsistencies through a combinatorial optimization scheme otherwise. Briefly, reads were first filtered to exclude reads in which there were more than three errors in the UMI sequence. For UMI sequences where each trimer contains at least two identical nucleotides, a majority vote was then performed to collapse the trimer into a monomer. If at least one trimer is inconclusive and contains three different nucleotides, we no longer treat each UMI sequence independently when collapsing trimers into monomers. Instead, we select one of the nucleotides in each trimer block to achieve maximal consistency between duplicates, that is to minimize the number of distinct collapsed UMI sequences. We formulate this task as a set cover problem for each gene as follows^31^. Let *S* be the set of sequenced homotrimer UMIs of a given gene (in a given cell). For $s\in S$ let *C*(*s*) denote the set of collapsed UMIs that can be obtained by combining single nucleotides that occur in each trimer block of *s*. Each such collapsed sequence $c\in C\left(s\right)$, for some $s\in S,$ can explain potentially multiple homotrimer UMIs ${s}^{{\prime} }$ if *c* is also contained in $C\left({s}^{{\prime} }\right)$. We therefore include one subset ${S}_{c}\subseteq S$ for each $c\in {\bigcup }_{\left\{s\in S\right\}}C\left(s\right)$ that contains all $s\in S$ for which $c\in C(s)$. The collection of sets ${S}_{c}$ of smallest cardinality that together include (‘cover’) all sequenced UMIs in *S* therefore corresponds to the smallest set of collapsed UMIs that explain all $s\in S$. To find this smallest set of collapsed UMIs, we use a greedy algorithm that starts from the empty set and in each iteration adds the subset *S*_*c*_ (that is, collapsed UMI *c*) that explains the largest number of yet unexplained sequenced UMIs. The solution returned by this algorithm is guaranteed to be within a logarithmic factor of the optimal solution^31^. In our experiments, the solution of the greedy approach was identical to the optimal solution for more than 90% of the genes. We computed the optimal solution using an integer linear programming approach, where decision variables model the inclusion or exclusion of sets *S*_*c*_ and linear inequalities enforce each sequenced UMI to be covered by at least one such set, that is to be explained by at least one collapsed UMI.

### Settings for simulated UMIs

We simulated UMI data of length 30 (ten blocks of nucleotide trimers) to test the accuracy of our UMI correction methodology by using the ResimPy tool. We mimicked the PCR amplification and sequencing errors seen with ONT sequencing, as this sequencing methodology suffers from indels and base calling errors more frequently than PacBio or Illumina sequencing. UMIs were generated following an approach that was first described by UMI-tools^11^. Briefly, we simulated homotrimer blocks of UMIs at random, with an amplification rate (-ampl_rate) ranging between 0.8 and 1.0 and then simulated PCR cycles so that each UMI was duplicated to the probability of amplification. PCR errors were then randomly added and assigned new probabilities of amplification. A predefined number of UMIs were randomly sampled to simulate sequencing depth and sequencing errors introduced with a specified probability. Finally, errors were detected by assessing the complementarity of homotrimers across the full UMI sequence. If no errors were detected, then the homotrimers were collapsed into single nucleotide bases. However, if errors were identified, then collapsing into single nucleotides was performed using the most common nucleotide within the trimer. If a most common nucleotide could not be determined, then a single nucleotide was selected at random for collapsing. The following values were used within our simulations. Sequencing depth 400; number of UMIs 50 (-umi_num); UMI length 12 (-umi_len); PCR error rate 3.6 × 10^−6^ (-seq_err); error rate 1 × 10^−1^–1 × 10^−7^ and number of PCR cycles 12 (-pcr_num); permutation tests 50 (-perm_num).

### ResimPy: simulating PCR artifacts in UMI-attached reads

We developed ResimPy for simulating UMI-attached reads. The total number of reads ${m}^{\left(i+1\right)}$ at PCR cycle $i+1$ comes from two sources: reads that are PCR amplified and those that are not. This can be described by a Galton–Watson branching process^32–34^ as follows

1 $${m}^{\left(i+1\right)}={m}^{\left(i\right)}+{n}^{(i)}$$

Here, ${n}^{(i)}$ is the number of reads to be amplified, determined by an amplification rate $\alpha$. According to Chen et al.^32^, ${n}^{(i)}$ follows a binomial distribution ${\mathrm{Binom}}({m}^{\left(i\right)},\alpha )$. The ${n}^{(i)}$ reads to be amplified are randomly selected from the set $\left\{\mathrm{1,2},\ldots ,{m}^{(i)}\right\}$ without replacement. This ensures that each read has an equal chance of being amplified. The same process applied for every two adjacent PCR cycles.

To simulate PCR errors, we implemented another Galton–Watson branching process. The total number of base errors ${u}^{(i+1)}$ at PCR cycle $i+1$ is modeled by:

2 $${u}^{\left(i+1\right)}={u}^{\left(i\right)}+{v}^{\left(i\right)}$$

Here, ${v}^{\left(i\right)}$ represents the number of base errors to be synthesized at PCR cycle $i+1$. Following Rabadan et al.^35^, ${v}^{\left(i\right)}$ is generated using a negative binomial distribution ${\mathrm{NBinom}}(r,q)$. Here *q* represents the probability of a base being successfully synthesized, which is derived by subtracting the base error probability *P*_e_ from 1 (that is $1-{P}_{\mathrm{e}}$). The variable *r* is determined by the product of the number of successfully synthesized bases calculated by $q\times {t}^{\left(i\right)}$ the positions of these ${v}^{\left(i\right)}$ PCR errors were randomly chosen from set $\left\{\mathrm{1,2},\ldots ,{t}^{(i)}\right\}$, where ${t}^{(i)}$ represents the total number of bases to be synthesized at PCR cycle *i* + 1. Finally, the base at each error sequence position was substituted by one of the remaining three types of bases, drawn from a discrete uniform distribution $U(\mathrm{1,3})$, where 1 and 3 represent the indices of the first and the third remaining bases, indicating that each one gains an equal chance for substitution. We use the same method to simulate sequencing errors. While simulation data provide some evidence for UMI deduplication performance, it is important to note that simulations can be biased. Therefore, we complement our simulations with experimentally derived data using our CMI approach described below.

### CMI and error evaluation in bulk sequencing

To measure the error rate and evaluate the accuracy of our UMIs following library preparation and sequencing, we synthesized a common sequence (GGGAAACCCTTTGGGCCCTTTAAACCCTTT) in replacement of a UMI to our polyA capture oligonucleotide. Following sequencing the CMI sequence was identified upstream of the SMART primer by pattern matching for GTACTCTGCGTTGATACCACTGCTT. The accuracy of our CMI was then determined by comparing the expected synthesized sequence to the extracted CMI sequence. The percentages of CMI that show full complementarity with the expected sequence were counted and the numbers of errors were determined for the inaccurate CMIs.

### Comparison between UMI-tools and homotrimer CMI deduplication methods

After mapping the reads to the reference genome at the gene level, we processed the data using two different strategies: UMI-tools and homotrimer deduplication. For homotrimer deduplication, we used the full length of the CMI sequence, while for UMI-tools we collapsed the CMI into a monomer by selecting the first base for each trimer block. The inclusion of the CMI sequence to our reads provides an experimental ground truth with which to evaluate the accuracy of each deduplication strategy. To assess the accuracy of the final deduplicated counts, we compared them to the expected ground truth CMI gene count of 1.

### 10X Chromium library preparation

We prepared a single-cell suspension using JJN3 and 5TGM1 cells using the standard 10X Genomics chromium protocol as per the manufacturer’s instructions. Briefly, cells were filtered into a single-cell suspension using a 40 μM Flomi cell strainer before being counted. We performed 10X Chromium library preparation following the manufacturer’s protocol. Briefly, we loaded 3,300 JJN3:5TGM1 cells at a 50/50 split into a single channel of the 10X Chromium instrument. Cells were barcoded and reverse transcribed into cDNA using the Chromium Single Cell 3′ library kit and get bead v.3.1. We performed ten cycles of PCR amplification before cleaning up the library using 0.6× SPRI Select beads. The library was split and a further 20 or 25 PCR cycles were performed using a biotin oligonucleotide (5-PCBio-CTACACGACGCTCTTCCGATCT) and then cDNA was enriched using Dynabeads MyOne streptavidin T1 magnetic beads (Invitrogen). The beads were washed in 2× binding buffer (10 mM Tric-HCL pH 7.5, 1 mM EDTA and 2 M NaCl), then samples were added to an equi-volume amount of 2× binding buffer and incubated at room temperature for 10 min. Beads were placed in a magnetic rack and then washed twice in 1× binding buffer. The beads were resuspended in H_2_O and incubated at room temperature and subjected to long-wave ultraviolet light (~366 nm) for 10 min. Magnetic beads were removed, and library was quantified using the Qubit High-sensitivity kit. Libraries were then prepared before sequencing.

### Drop-seq library preparation

Single-cell capture and reverse transcription were performed as previously described^20^. Briefly, JJN3 and 5TGM1 cells (20:80 ratio) were filtered into a single-cell suspension using a 40 μM Flomi cell strainer before being counted. Cells were loaded into the DolomiteBio Nadia Innovate system at a concentration of 310 cells per μl. Custom synthesized beads were loaded into the microfluidic cartridge at a concentration of 620,000 beads per ml. Cell capture was then performed using the standard Nadia Innovate protocol according to the manufacturer’s instructions. The droplet emulsion was then incubated for 10 min before being disrupted with 1*H*,1*H*,2*H*,2*H*-perfluoro-1-octanol (Sigma) and beads were released into aqueous solution. After several washes, the beads were subjected to reverse transcription. Before PCR amplification, beads were treated with ExoI exonuclease for 45 min. PCR amplification was then performed using the SMART PCR primer (AAGCAGTGGTATCAACGCAGAGT) and cDNA was subsequently purified using AMPure beads (Beckman Coulter). The library was split and a further 20 or 25 PCR cycles^20^ were performed using a biotin oligonucleotide (5-PCBio-TACACGACGCTCTTCCGATCT) and then cDNA was enriched using Dynabeads MyOne streptavidin T1 magnetic beads (Invitrogen). The beads were washed in 2× binding buffer (10 mM Tric-HCL pH 7.5, 1 mM EDTA and 2 M NaCl) then samples were added to an equi-volume amount of 2× binding buffer and incubated at room temperature for 10 mins. Beads were placed in a magnetic rack and then washed with twice with 1× binding buffer. The beads were resuspended in H_2_O and incubated at room temperature and subjected to long-wave ultraviolet light (~366 nm) for 10 min. Magnetic beads were removed, and library was quantified using the Qubit High-sensitivity kit. Libraries were then prepared for sequencing.

### Bulk and single-cell library preparation and ONT sequencing

A total of 500 ng of single-cell PCR input was used as a template for ONT library preparation. Library preparation was performed using the SQK-LSK114 (kit V14) ligation sequencing kit, following the manufacturer’s protocol. Samples were then sequenced on either a Flongle device or a PromethION device using R10.4 (FLO-PRO114M) flow cells.

### 10X analysis workflow

We performed base calling on the raw fast5 data to generate fastq files using Guppy (v.6.4.8) (guppy_basecaller –compress-fastq -c dna_r10.4_e8.1_sup.cfg -x ‘cuda:0’) in GPU mode from ONT running on a RTX3090 graphics card. To process the 10X chromium data, we wrote a custom cgatcore pipeline (https://github.com/cribbslab/TallyTriN/blob/main/tallytrin/pipeline_10x.py)^22^. We first determined the orientation of the reads and if a poly-T sequence was detected we reverse complemented the read. Next, we identified the barcode and UMI based on the pairwise alignment of the sequence AGATCGGAAGAGCGT and AAAAAAAAA and identified the sequence between these alignments. We next removed reads that were greater or equal to 28 bp and isolated the barcode as the first 16 bp and the UMI the following 12 bp. The barcode and UMI sequence were then appended to the name of the fastq read using the underscore delimiter. Next, to remove barcode errors we parsed the barcodes from each read in the fastq file and then selected the most common barcode sequences using the number of expected cells in our library as the threshold. Next, for every read in the fastq file we then identified the closest barcode match for each read, allowing for two mismatches. Mapping was performed using minimap2 (v.2.25)^36^, with the following settings: -ax splice -uf –MD –sam-hit-only –junc-bed and using the reference transcriptome for human hg38 and mouse mm10. The resulting .bam file was sorted and indexed before adding the transcript name to the XT tag within the .bam file. Counting was then performed using UMI-tools –method=directional before being converted to a market matrix format. Raw transcript expression matrices generated by UMI-tools count were processed using R/Bioconductor (v.4.3.0), the raw market matrix files were imported into R using bustools (v.0.42.0) and the Seurat^37,38^ package (v.4.3.0). Transcript matrices were cell-level scaled and log-transformed. The top 2,000 highly variable genes were then selected based on variance stabilizing transformation that was used for principal component analysis. Clustering was performed within Seurat using the Louvain algorithm. To visualize the single-cell data, we projected data onto a uniform manifold approximation and projection (UMAP)^39^.

### Drop-seq analysis workflow

We performed base calling on the raw fast5 data to generate fastq files using Guppy (v.6.4.8) (guppy_basecaller –compress-fastq -c dna_r10.4_e8.1_sup.cfg -x ‘cuda:0’) in GPU mode from ONT running on a RTX3090 graphics card. To process the drop-seq data, we wrote a custom cgatcore pipeline (https://github.com/cribbslab/TallyTriN)^22^. We followed the workflow previously described for identifying barcodes and UMIs using scCOLOR-seq sequencing analysis^13^. Briefly, to determine the orientation of our reads, we first searched for the presence of a polyA sequence or a poly-T sequence. In cases where the poly-T was identified, we reverse complemented the read. We next identified the barcode sequence by searching for the polyA region and flanking regions before and after the barcode. The trimer UMI was identified based on the primer sequence GTACTCTGCGTT at the TSO distal end of the read, allowing for two mismatches. Barcodes and UMIs that had a length less than 48 base pairs were filtered. To conduct monomer-based analyses, a random base was selected from each homotrimer in the UMI or CMI and collapsed into a monomer. Homotrimer UMI correction was performed following mapping using minimap2 (v.2.25)^36^. Mapping settings were as follows: -ax splice -uf –MD –sam-hit-only –junc-bed and using the reference transcriptome for human hg38 and mouse mm10. The resulting .sam file was sorted and indexed using samtools^25^. For monomer UMI, counting was performed using UMI-tools before being converted to a market matrix format. For homotrimer UMI correction, the counting was performed using the script greedy.py within the TallyTriN repository. Raw transcript expression matrices generated by UMI-tools count and greedy.py were processed using R and Bioconductor (v.4.3.0) and custom scripts were used to generate barnyard plots showing the proportion of mouse and human cells. Transcript matrices were cell-level scaled and center log ratio transformed. The top 3,000 highly variable genes were then selected based on variance stabilizing transformation that was used for principal component analysis. Clustering was performed within Seurat using the Louvain algorithm. To visualize the single-cell data, we projected data onto a UMAP^39^.

### Reporting summary

Further information on research design is available in the Nature Portfolio Reporting Summary linked to this article.

## Online content

Any methods, additional references, Nature Portfolio reporting summaries, source data, extended data, supplementary information, acknowledgements, peer review information; details of author contributions and competing interests; and statements of data and code availability are available at 10.1038/s41592-024-02168-y.

### Supplementary information

Supplementary Information Supplementary Figs. 1–19.

Reporting Summary

Supplementary Tables Supplementary Tables 1–5.

## Data Availability

Sequencing data have been deposited to the Gene Expression Omnibus under the accession number GSE218899. All analysis was performed using hg38 ensembl 98 version.

## Code availability

Source data are provided with this paper. All custom pipelines used within this analysis are available on GitHub (https://github.com/cribbslab/TallyTriN). ResimPy (v.0.0.1) is available on GitHub (https://github.com/cribbslab/resimpy).

## References

[1] Hug H, Schuler R (2003). Measurement of the number of molecules of a single mRNA species in a complex mRNA preparation. J. Theor. Biol..

[2] Aird D (2011). Analyzing and minimizing PCR amplification bias in Illumina sequencing libraries. Genome Biol..

[3] Kivioja T (2011). Counting absolute numbers of molecules using unique molecular identifiers. Nat. Methods.

[4] Islam S (2014). Quantitative single-cell RNA-seq with unique molecular identifiers. Nat. Methods.

[5] Hagemann-Jensen M (2020). Single-cell RNA counting at allele and isoform resolution using Smart-seq3. Nat. Biotechnol..

[6] Schmitt MW (2012). Detection of ultra-rare mutations by next-generation sequencing. Proc. Natl Acad. Sci. USA.

[7] Kukita Y (2015). High-fidelity target sequencing of individual molecules identified using barcode sequences: de novo detection and absolute quantitation of mutations in plasma cell-free DNA from cancer patients. DNA Res..

[8] Peng X, Dorman KS (2023). Accurate estimation of molecular counts from amplicon sequence data with unique molecular identifiers. Bioinformatics.

[9] Shafin K (2021). Haplotype-aware variant calling with PEPPER-Margin-DeepVariant enables high accuracy in nanopore long-reads. Nat. Methods.

[10] You, Y. et al. Identification of cell barcodes from long-read single-cell RNA-seq with BLAZE. *Genome Biol.* **24**, 66 (2023). 10.1186/s13059-023-02907-y PMC10077662 37024980

[11] Smith T, Heger A, Sudbery I (2017). UMI-tools: modeling sequencing errors in Unique Molecular Identifiers to improve quantification accuracy. Genome Res..

[12] Volden R, Vollmers C (2022). Single-cell isoform analysis in human immune cells. Genome Biol..

[13] Philpott, M. et al. Nanopore sequencing of single-cell transcriptomes with scCOLOR-seq. *Nat. Biotechnol*. **39**, 1517–1520 (2021). 10.1038/s41587-021-00965-w PMC8668430 34211161

[14] Karst SM (2021). High-accuracy long-read amplicon sequences using unique molecular identifiers with Nanopore or PacBio sequencing. Nat. Methods.

[15] Tsagiopoulou M (2021). UMIc: a preprocessing method for UMI deduplication and reads correction. Front. Genet..

[16] Bose S (2015). Scalable microfluidics for single-cell RNA printing and sequencing. Genome Biol..

[17] Shagin DA (2017). A high-throughput assay for quantitative measurement of PCR errors. Sci. Rep..

[18] Potapov V, Ong JL (2017). Examining sources of error in PCR by single-molecule sequencing. PLoS ONE.

[19] Pflug FG, von Haeseler A (2018). TRUmiCount: correctly counting absolute numbers of molecules using unique molecular identifiers. Bioinformatics.

[20] Macosko EZ (2015). Highly parallel genome-wide expression profiling of individual cells using nanoliter droplets. Cell.

[21] Zhu YY, Machleder EM, Chenchik A, Li R, Siebert PD (2001). Reverse transcriptase template switching: a SMART approach for full-length cDNA library construction. Biotechniques.

[22] Cribbs A (2019). CGAT-core: a python framework for building scalable, reproducible computational biology workflows [version 1; peer review: 1 approved, 1 approved with reservations]. F1000 Res..

[23] FastQC: a quality control tool for high throughput sequence data (Brabham Bioinformatics, 2010).

[24] Ewels P, Magnusson M, Lundin S, Kaller M (2016). MultiQC: summarize analysis results for multiple tools and samples in a single report. Bioinformatics.

[25] Li H (2009). The Sequence Alignment/Map format and SAMtools. Bioinformatics.

[26] Liao Y, Smyth GK, Shi W (2014). featureCounts: an efficient general purpose program for assigning sequence reads to genomic features. Bioinformatics.

[27] Love MI, Huber W, Anders S (2014). Moderated estimation of fold change and dispersion for RNA-seq data with DESeq2. Genome Biol..

[28] Cribbs A (2019). CGAT-core: a python framework for building scalable, reproducible computational biology workflows [version 2; peer review: 1 approved, 1 approved with reservations]. F1000 Res..

[29] Kim D, Paggi JM, Park C, Bennett C, Salzberg SL (2019). Graph-based genome alignment and genotyping with HISAT2 and HISAT-genotype. Nat. Biotechnol..

[30] Liao Y, Smyth GK, Shi W (2013). The Subread aligner: fast, accurate and scalable read mapping by seed-and-vote. Nucleic Acids Res..

[31] Chvatal V (1979). A greedy heuristic for the set-covering problem. Math. Oper. Res..

[32] Chen YJ (2020). Quantifying molecular bias in DNA data storage. Nat. Commun..

[33] Lalam, N. Statistical inference for quantitative polymerase chain reaction using a hidden Markov model: a Bayesian approach. *Stat. Appl. Genet. Mol. Biol.* **6**, 10 (2007). 10.2202/1544-6115.1253 17402916

[34] Wagner A (1994). Surveys of gene families using polymerase chain-reaction—PCR selection and PCR drift. Syst. Biol..

[35] Rabadan R (2018). On statistical modeling of sequencing noise in high depth data to assess tumor evolution. J. Stat. Phys..

[36] Li H (2018). Minimap2: pairwise alignment for nucleotide sequences. Bioinformatics.

[37] Stuart T (2019). Comprehensive integration of single-cell data. Cell.

[38] Hao Y (2021). Integrated analysis of multimodal single-cell data. Cell.

[39] McInnes, L., Healy, J., Saul, N. & Großberger, L. UMAP: uniform manifold approximation and projection. *J. Open Source Softw.* **3**, 861 (2018).

