# Supplementary material for: Correcting PCR amplification errors in unique molecular identifiers to generate accurate numbers of sequencing molecules
Source: Nat Methods. 2024 Feb 5;21(3):401–5. doi: 10.1038/s41592-024-02168-y (PMC10927542; doi:10.1038/s41592-024-02168-y)
Supplement: Supplementary file 1 — Supplementary Figs. 1–19. [file 41592_2024_2168_MOESM1_ESM.pdf]

nature methods

Brief Communication

https://doi.org/10.1038/s41592-024-02168-y

Correcting PCR amplification errors in unique molecular identifiers to generate accurate numbers of sequencing molecules

In the format provided by the
authors and unedited

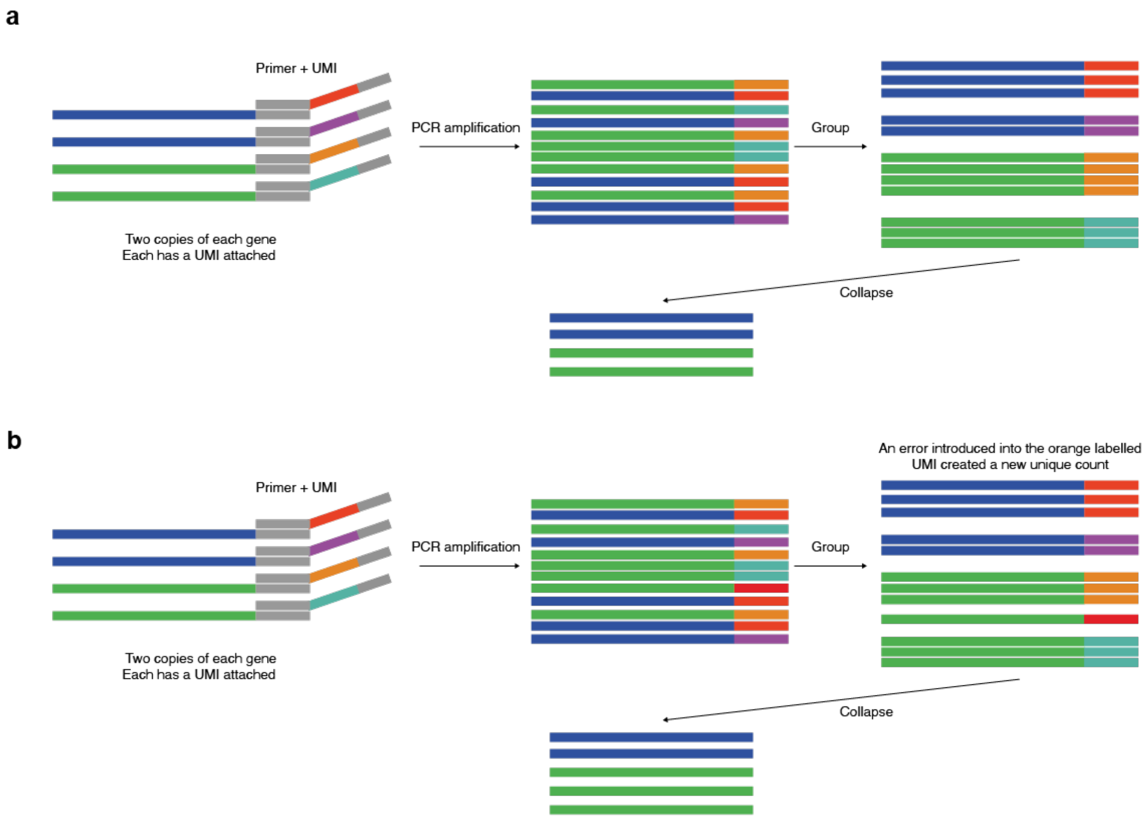

Supplementary Figure 1: UMI errors from sequencing or PCR increase UMI counting.
a, Ideal UMI collapsing example: This figure illustrates an ideal scenario where transcripts (two blue and two green) are labelled with unique molecular identifier barcodes (UMIs) and PCR amplification is performed. Due to PCR amplification bias, longer transcripts have a lower amplification rate than shorter ones. The sequenced reads are then grouped together based on the set of UMIs and then collapsed within those groups to match the original number of transcripts. **b**, Increased UMI counting occurs due to errors: In real situations, errors occur during PCR amplification and sequencing, which can lead to increased UMI counts. As shown in this example, an error occurs within one of the UMIs which results in a higher count of unique UMIs than the actual number of input transcripts in the final library. This phenomenon can affect downstream analysis and should be taken into consideration otherwise they will lead to false positives when performing differential expression analysis.

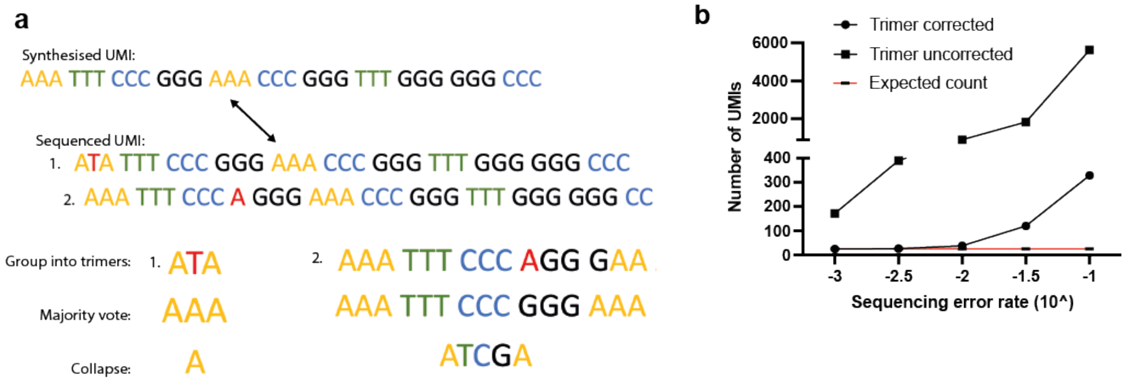

Supplementary Figure 2: Improved UMI deduplication using homotrimer blocks of nucleotides.

a, Homotrimer blocks of nucleotides are used to synthesise UMIs, which enables efficient error correction through a majority vote between the trimer blocks. This approach does not require knowledge of the original oligonucleotide, and the trimers are resilient to base pair errors (example 1) and insertions/deletions (example 2). Errors are removed before collapsing the sequence to a single-base and then performing downstream analyses. **b**, We simulated UMIs with increasing error rates were modelled the correction of trimer sequences using the majority vote approach as described in **a**. To handle homotrimer errors more robustly, we subsequently developed a new model, which is described within the methods section "homotrimer correction". Error bars in b are plotted but not visible because the CVs are very small over three different repeated simulations. Error bars represent the mean and +/- Standard Deviation.

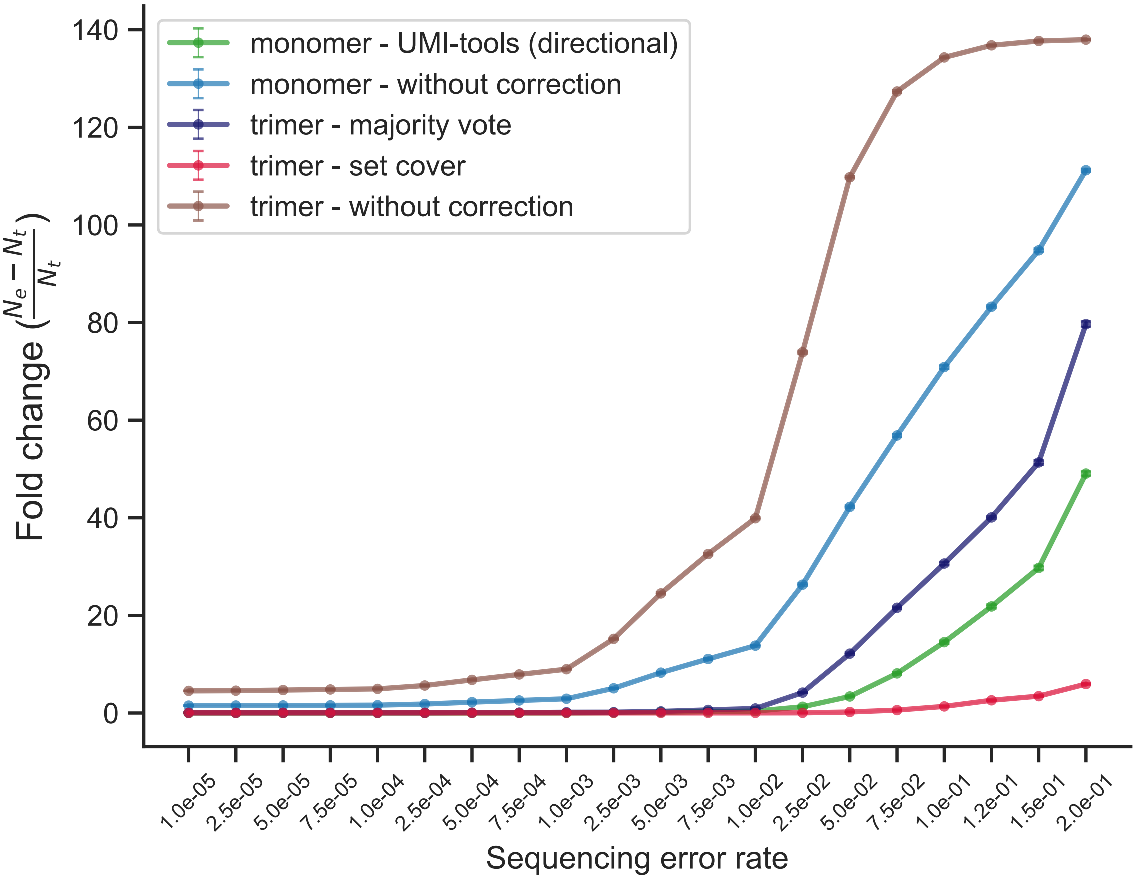

Supplementary Figure 3: Comparison of homotrimer and monomer UMI for demultiplexing.

We evaluated the effectiveness of homotrimer UMIs versus monomer UMIs for demultiplexing by simulating base UMIs with increasing error rates and calculated the fold change between the ground truth and the output after computational error correction is applied. Our findings demonstrate that the performance of correcting homotrimer UMIs using the set coverage approach outperform that of uncorrected monomer UMIs and correcting monomer UMIs using computational methods. PCR cycles were set to 12. Sequencing depth was set to 400. The number of initial molecules was set to 50. The length of homotrimer UMIs was set to 36bp. *Ne* represents the number of sequenced molecules. *Nt* represents the number of initial molecules. Error bars are s.d. of 3 independent simulations.

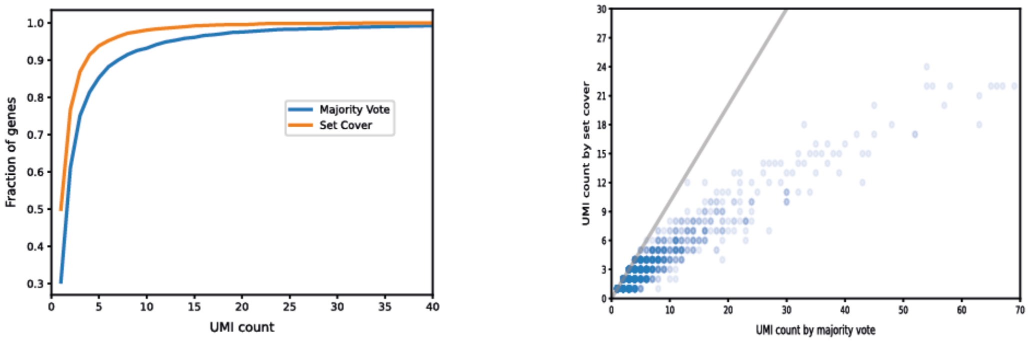

Supplementary Figure 4: The majority vote method is improved using a set coverage solution.

a, A cumulative plot showing the fraction of genes that have been collapsed to a common molecular identifier (CMI) count of less than or equal to the value shown on the x-axis by either the majority vote approach or the set cover-based optimization method. A CMI refers to a common homotrimer sequence attached to every captured RNA molecule, when demultiplexed without errors, the RNA count will equal 1, any errors will inflate the RNA molecule count. The data used for this approach is ONT sequencing using R9.4 and LSK110 chemistry. Only genes with at least 2 mapped reads were considered (n=3,428). Maximal UMI counts returned by majority vote and set cover optimization are 245 and 72, respectively. **b**, A scatter plot comparing UMI counts obtained using the majority vote approach (x-axis) to counts returned by the greedy set cover algorithm (y-axis). Only genes with at least 2 mapped reads were considered (n=3,428). To simplify visualization, we excluded genes with large majority counts (x,y) = (83, 22), (91, 24), (96, 31), (106, 32), (113, 33) and (245, 72).

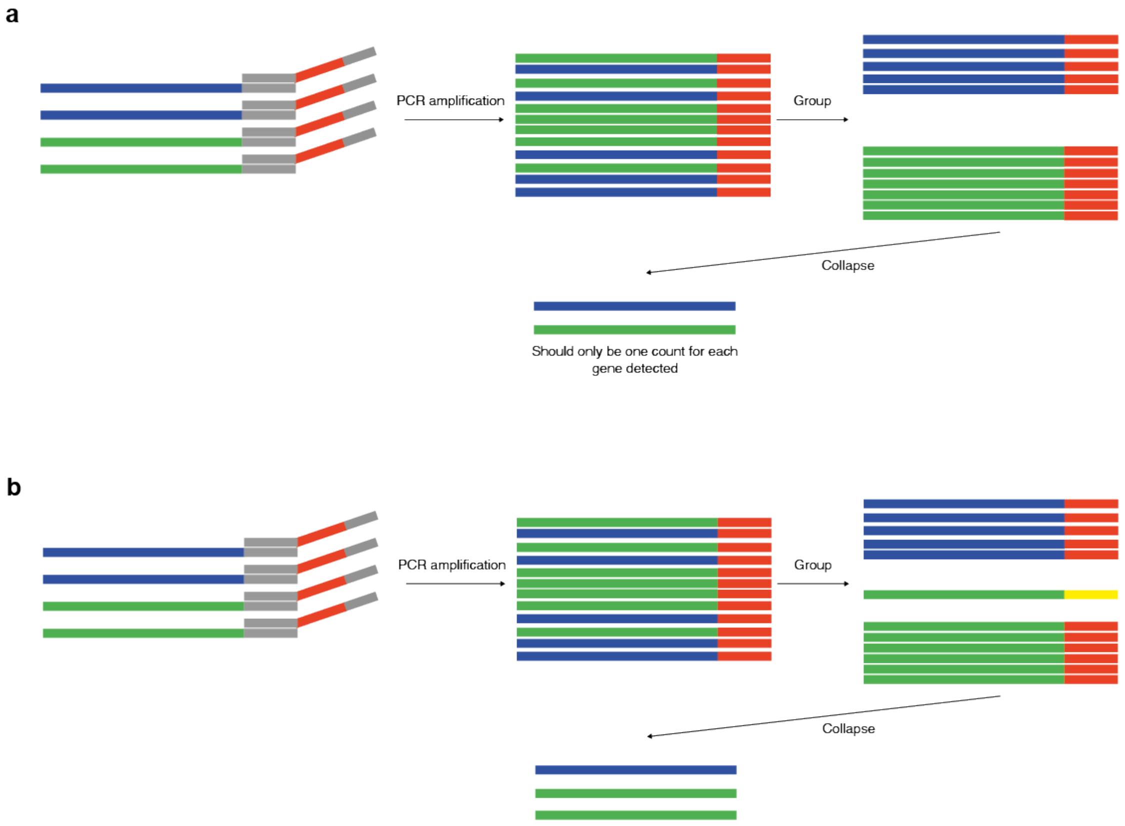

Supplementary Figure 5: Empirical evaluation of transcript counting with Common Molecular Identifiers (CMIs).

a, An Ideal CMI collapsing example: In this scenario, transcripts (two blue and two green) are labelled with a common molecular identifier barcode (CMIs; labelled as red) and amplified via PCR. During transcripts grouping, all transcripts are labelled with the same common sequence. Therefore, following demultiplexing, each transcript should receive a count of one for every instance of detection. **b**, Increased counts result from the introduction of errors: This figure illustrates the effect of the errors within the CMI sequence. Any error introduced during PCR or sequencing creates a new CMI (labelled as yellow), resulting in an increase in transcript counts. This allows empirical evaluation of the effect of errors on the counting of transcripts, providing valuable insights into the accuracy of transcript quantification.

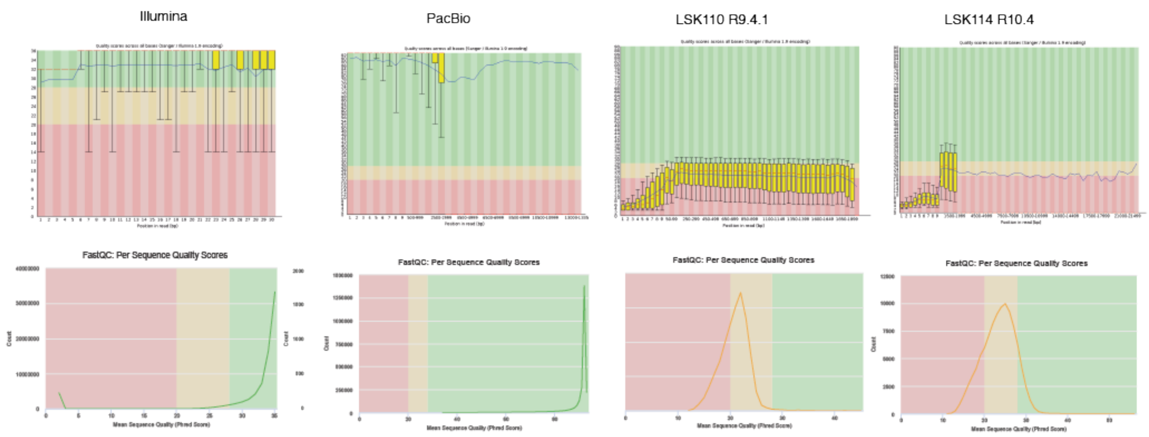

Supplementary Figure 6: Read quality control following sequencing by Illumina, PacBio and ONT.

The read quality outputs from FASTQC for Illumina, PacBio and ONT (old chemistry: LSK110 R9.4.1 and new kit14 chemistry LSK114 R10.4). Error bars are defined by FASTQC and the central red line is the median value, the yellow box represents the inter-quartile range (25-75%), the upper and lower whiskers represent the 10% and 90% points, the blue line represents the mean quality.

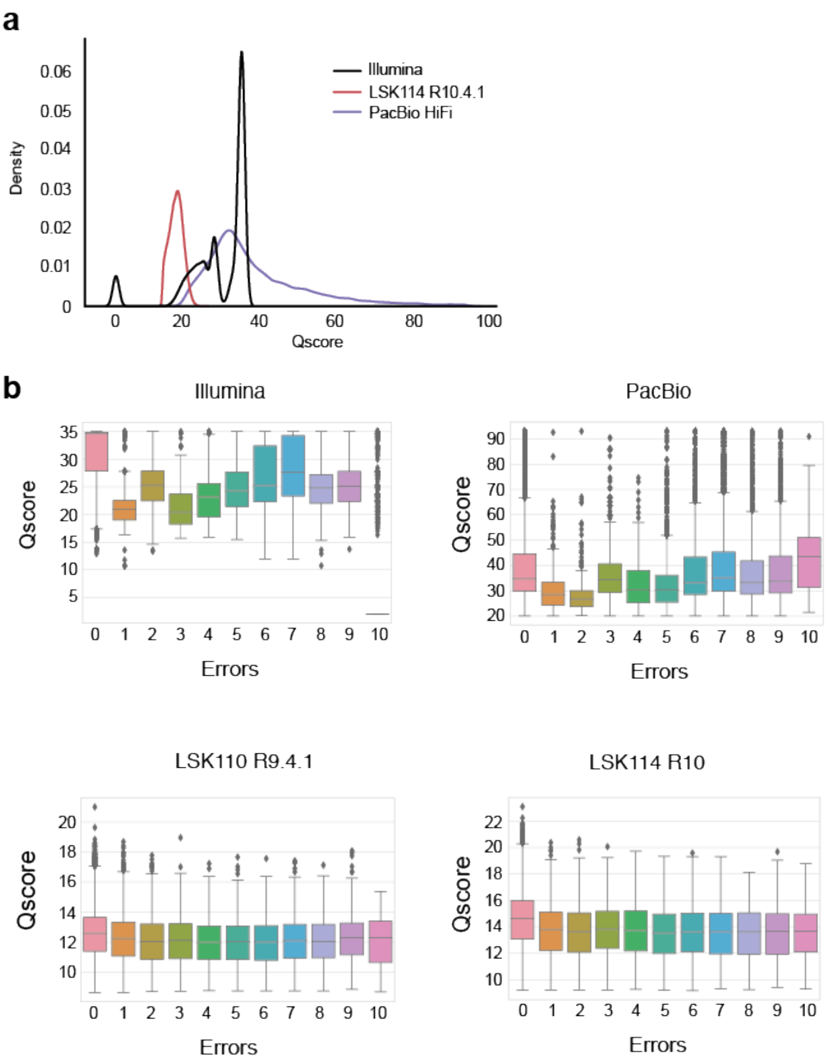

Supplementary Figure 7: Quality score following sequencing by Illumina, PacBio and ONT. The Qscore of each read as a relationship between the number of errors measured within the CMI, sequencing across Illumina, PacBio and ONT (old chemistry: LSK110 R9.4.1 and new kit14 chemistry LSK114 R10.4) technologies. **a**, Qscore represented as a density plot for the different sequencing platforms. **b**, The Qscore relationship with the number of errors detected within the CMI across the different sequencing platforms. The Error bars are plotted so that the central line is the median value, the box represents the inter-quartile range (25-75%), the upper and lower whiskers represent the 10% and 90% points, the points represent the extreme outliers.

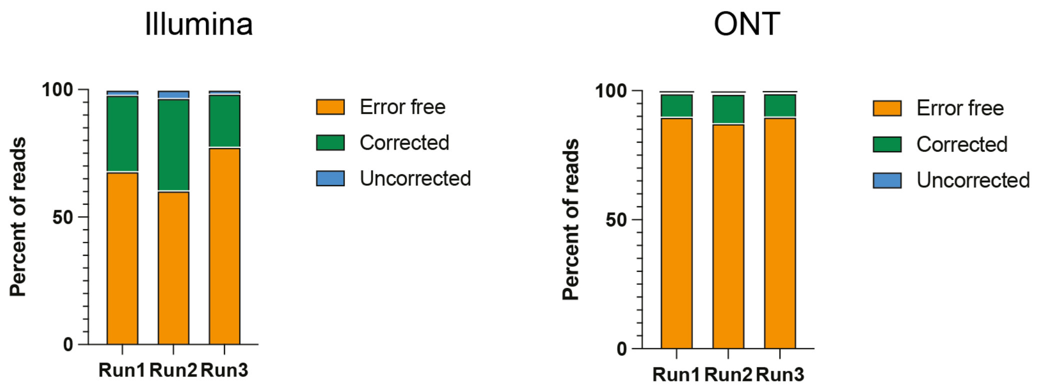

Supplementary Figure 8: Percent of correctly sequenced CMIs across Illumina and ONT sequencing platforms

The left panel shows the individual repeats that were performed following Illumina sequencing of a homotrimeric CMI tagged cDNA. Each run was performed independently and sequenced using separate flow cells. The right panel shows the same cDNA sequenced using the ONT platform, across three separate minION flow cells.

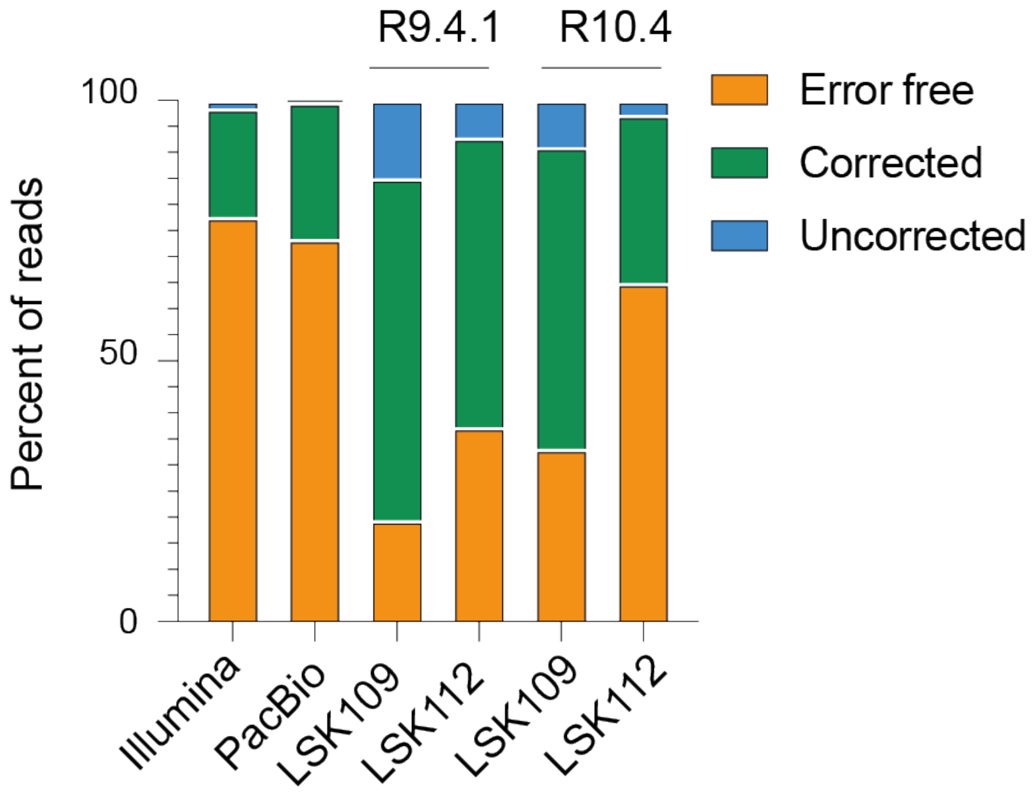

Supplementary Figure 9: Evaluation of CMI accuracy across Illumina, PacBio and legacy ONT chemistry.

The accuracy of sequencing was evaluated for legacy ONT chemistry, we measured the percentage of CMIs with a Hamming distance between the expected and the sequenced CMI. The results are shown for Illumina, PacBio, and ONT legacy chemistry sequencing. Data from Illumina and PacBio is the same as in Fig. 1h. Data from Illumina and ONT were performed in triplicate, whereas PacBio was performed as a single run.

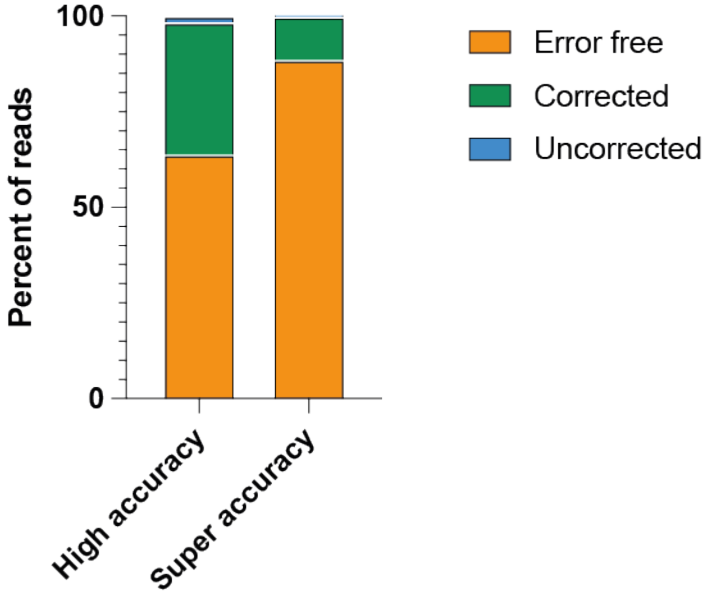

Supplementary Figure 10: Improved basecalling accuracy using super accuracy guppy basecalling for ONT technology.
Percent of CMIs that are correctly sequenced and then error corrected using homotrimer correction using either high accuracy guppy basecalling or super accuracy basecalling for the LSK114 chemistry and R10.4 flow cells.

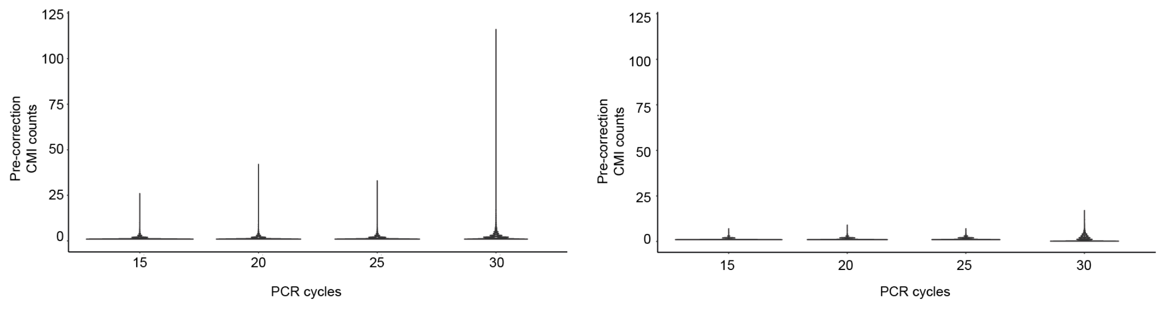

Supplementary Figure 11: CMI counts pre and post majority vote corrected.
The left panel shows a violin plot of the counts for each transcript pre majority vote correction following 15, 20, 25 and 30 PCR cycles. The right-hand panel shows the CMI counts post majority vote correction. The ground truth count for each transcript should be equal to 1, any counts above this indicate an error.

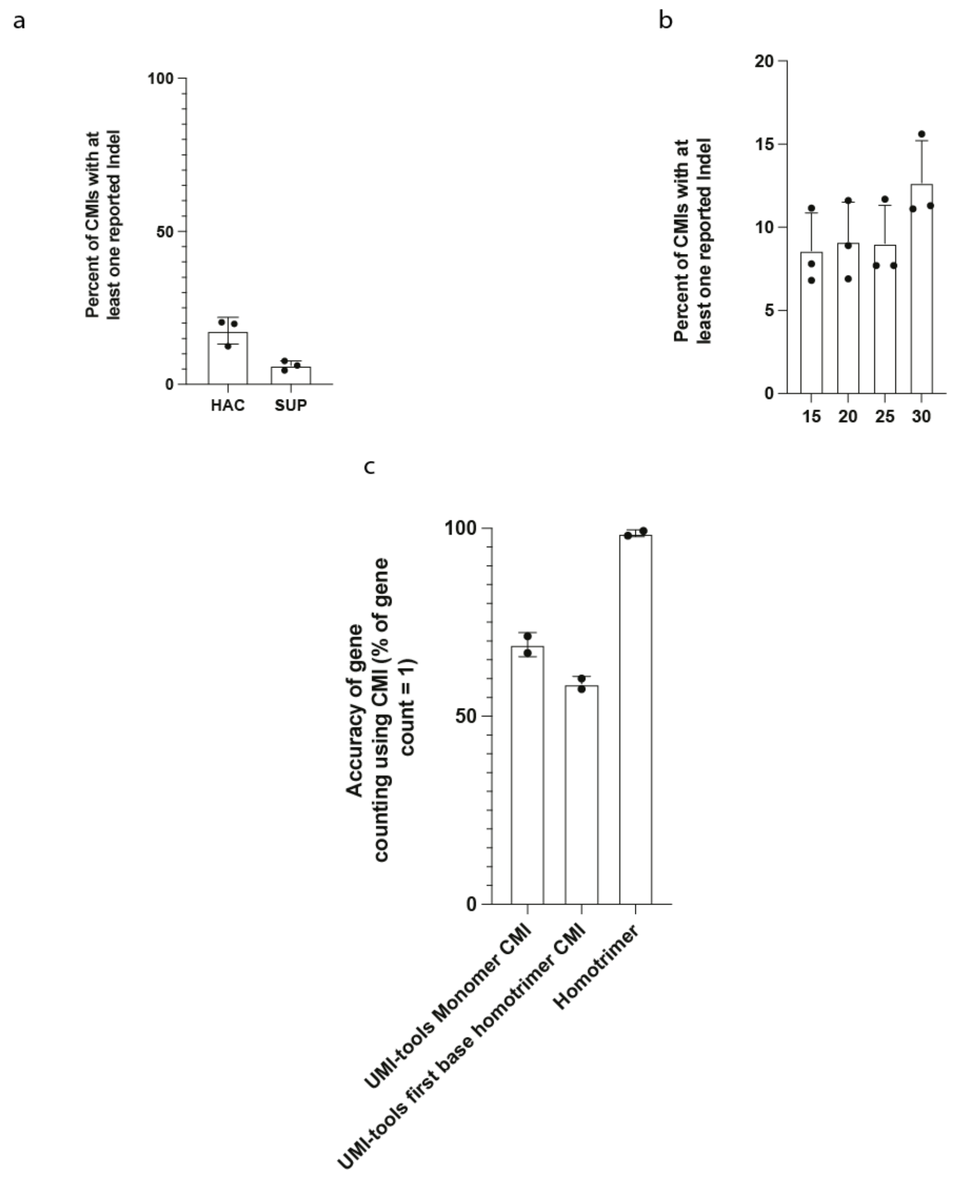

Supplementary Figure 12: The indel frequency and accuracy of counting using different CMI approaches.

a, The percent of CMIs with at least one indel were calculated for a bulk homotrimer CMI sequencing experiment using both High accuracy (HAC) and Super accuracy basecalling (SUP). **b,** The percent of CMIs with at least one indel were calculated for a bulk homotrimer CMI sequencing following 15, 20, 25 and 30 PCR cycles. **c,** This figure represents the percentage of genes with accurately quantified CMI after applying ONT sequencing. Three strategies were compared: (1) counting using a monomer-synthesised CMI and subsequent application of UMI-tools, (2) employing a homotrimer-synthesised CMI with a selection of the first base in the trimer block and (3) using the homotrimer error correction. Error bars are s.d. of 3 independent experiments.

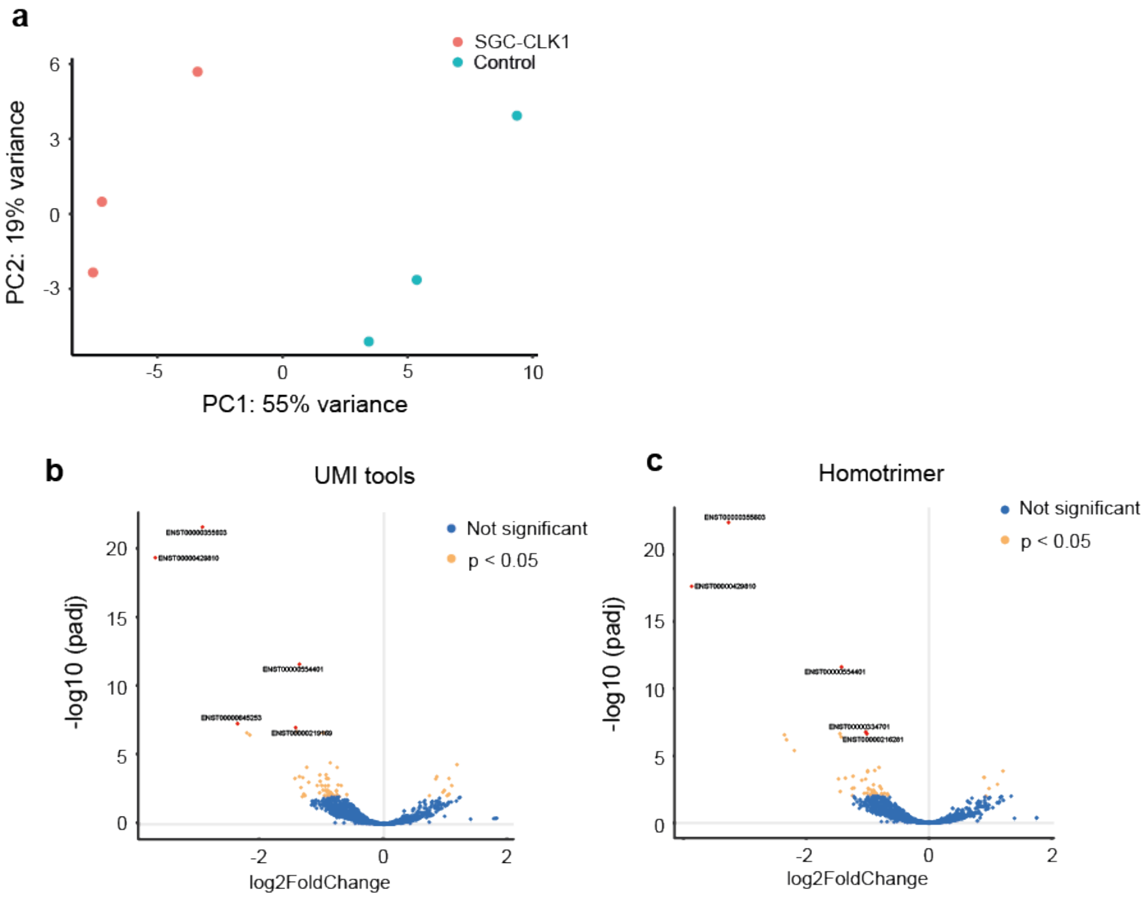

Supplementary Figure 13: Analysis of differential gene expression in RM82 Ewing's sarcoma cells treated with DMSO and CLK-1 inhibitor and sequenced using the ONT platform.

a, A PCR plot showing the variance for cells treated with either DMSO or CLK-1 inhibitor. **b**, A volcano plot showing the log2 fold change and -log10 padj values for cells treated with DMSO or CLK-1 inhibitor, analysed without the inclusion of a UMI during analysis. **d**, A volcano plot showing the log2 fold change and -log10 padj values for cells treated with DMSO or CLK-1 inhibitor and analysed using the homotrimer corrected UMI. These results demonstrate the utility of homotrimer correction in identifying differentially expressed genes and removal of false positive transcripts. Differential expression was determined using DESEq2 using a Wald test and a p adjusted value of < 0.05 was used as a threshold.

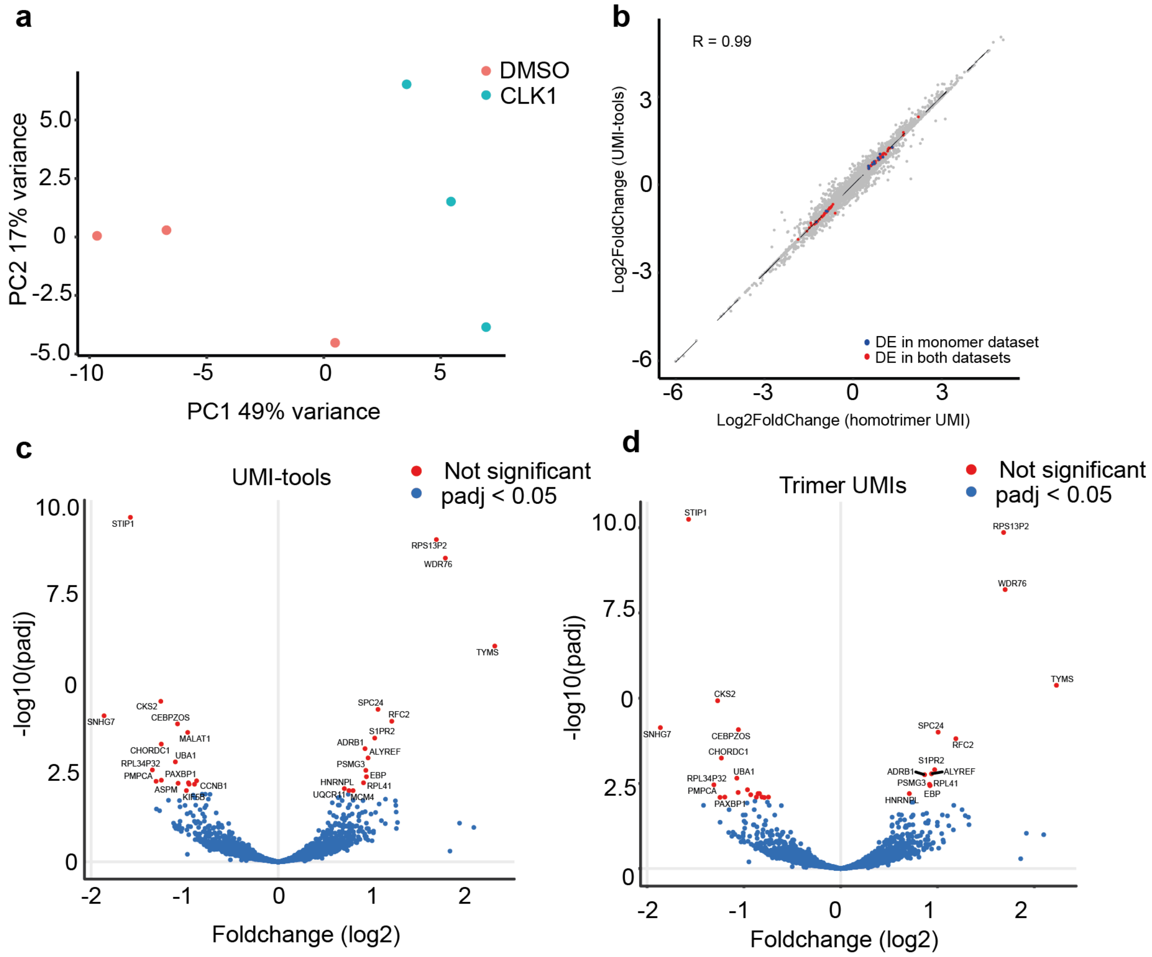

Supplementary Figure 14: Analysis of differential gene expression in RM82 Ewing's sarcoma cells treated with DMSO and CLK-1 inhibitor and sequenced using the Illumina platform.

a, A PCR plot showing the variance for cells treated with either DMSO or CLK-1 inhibitor. **b**, This scatter plot compares the log2 fold changes obtained from randomly collapsing each sequenced trimer UMI with those obtained from homotrimer UMI correction. **c**, A volcano plot showing the log2 fold change and -log10 padj values for cells treated with DMSO or CLK-1 inhibitor, analysed without the inclusion of a UMI during analysis. **d**, A volcano plot showing the log2 fold change and -log10 padj values for cells treated with DMSO or CLK-1 inhibitor and analysed using the homotrimer corrected UMI. These results demonstrate the utility of homotrimer correction in identifying differentially expressed genes and removal of false positive genes. Differential expression was determined using DESEq2 using a Wald test and a p adjusted value of < 0.05 was used as a threshold.

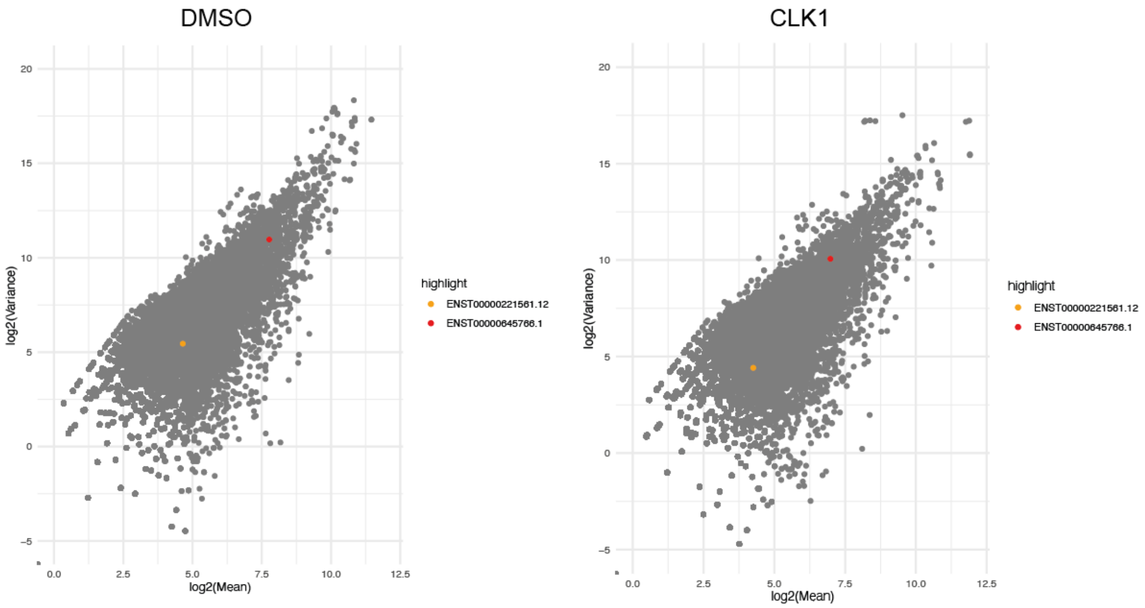

Supplementary Figure 15: Mean variance plot of transcripts showing samples treated with DMSO and CLK1.

The mean variance plots show that the transcript expression is correlated, with the higher the transcript expression the higher the variance. The data follows a negative binomial distribution. Two transcripts plotted in Fig. 1m-n are plotted as red and orange points.

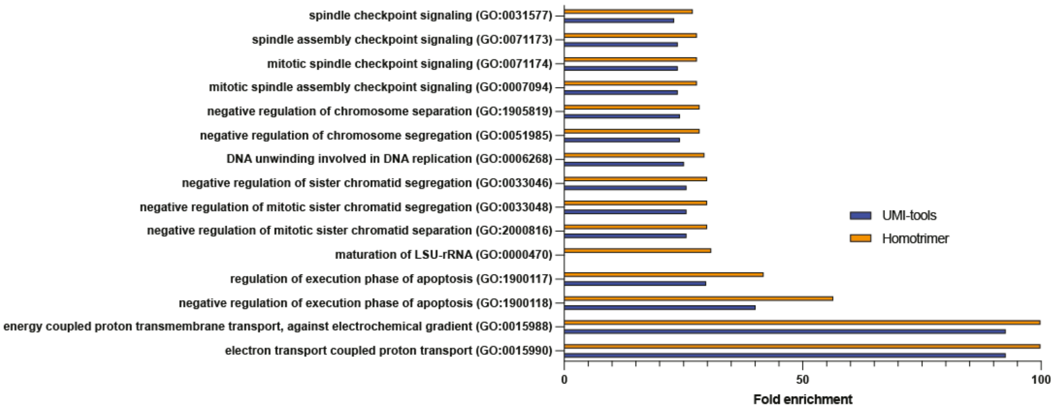

Supplementary Figure 16: GO analysis of the differentially expressed genes between DMSO and CLK-1 inhibitor.

Go analysis was performed for data shown in Fig. 1l that corrected using UMI-tools (blue bars) and differentially regulated genes following homotrimer correction (orange bars).

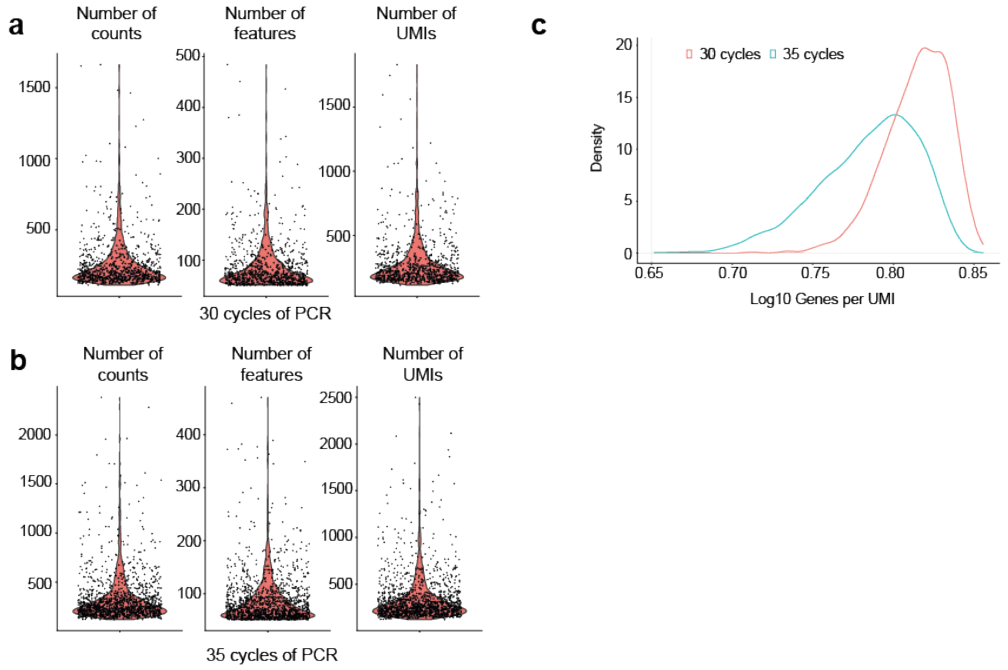

Supplementary Figure 17: Quality metrics for 10X chromium single-cell sequencing libraries amplified using 20 and 25 cycles of PCR.

The number of counts, features and number of UMIs for 10X Chromium libraries PCR amplified for 20 cycles (**a**) and 25 cycles (**b**). Each dot represents a single-cell following filtering. **c**, The log10 genes per UMI plotted as a density.

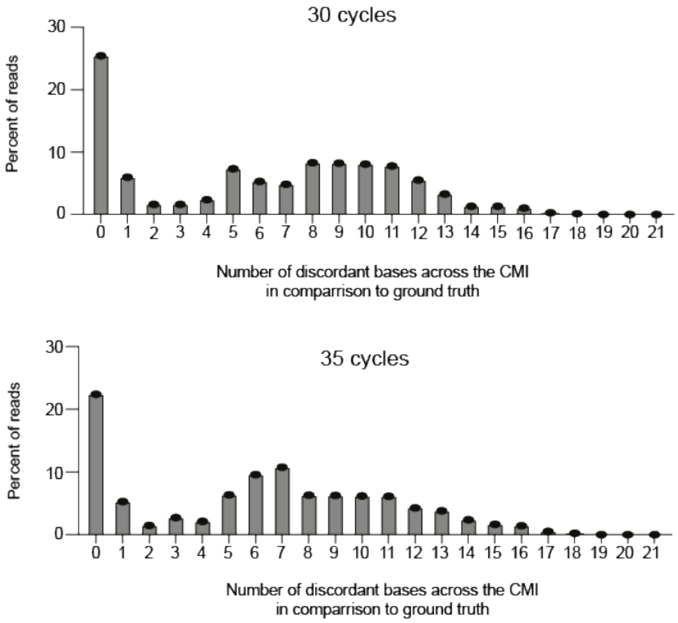

Supplementary Figure 18: The number of discordant bases across the full length of the homotrimer UMI

The number of errors per read between the sequenced CMI and the ground truth CMI following 30 and 35 PCR cycles. Error bars are s.d. of 3 independent experiments.

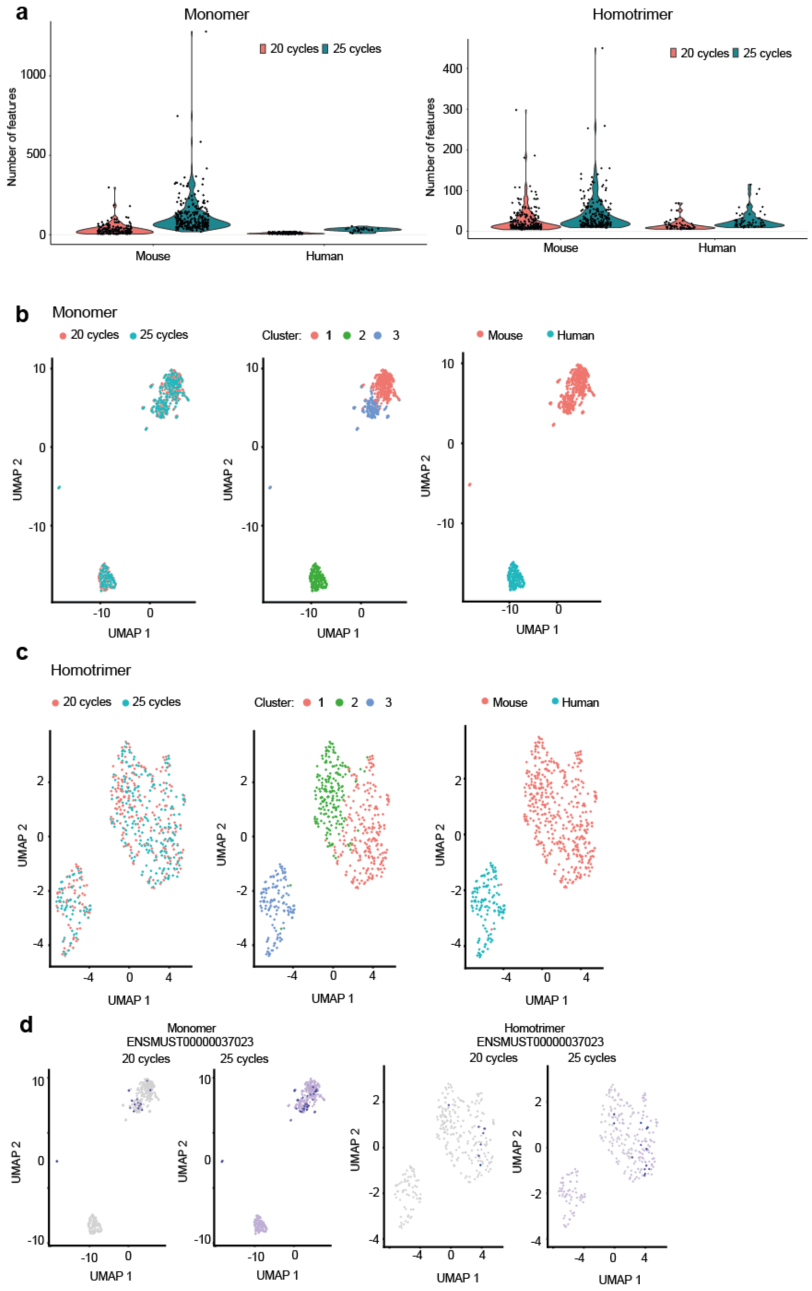

Supplementary Figure 19: Number of features and UMAP clustering for the integrated analysis of homotrimer drop-seq UMIs following 20 and 25 cycles of PCR.

a, The number of features detected within the mouse and human cells for monomer (left panel) and homotrimer (right panel) UMIs following 20 and 25 PCR cycles. UMAP plots showing the integration, clustering and annotation of libraries amplified following 20 and 25 PCR cycles for monomer (**b**) and homotrimer (**c**) UMIs. **d**, UMAP plots showing the expression of a non-significant gene ENSMUST0000037023 in monomers (left panels) and homotrimer corrected (right panels) following 20 and 25 cycles of PCR. Even though this gene is not considered significantly different between 20 and 25 cycles in both the monomer and homotrimer datasets, there is generally an overall increase in background counts following 25 cycles of PCR in the monomer dataset that is not apparent within the homotrimer dataset.
