# Supplementary material for: Correcting PCR amplification errors in unique molecular identifiers to generate accurate numbers of sequencing molecules
Source: Nat Methods. 2024 Feb 5;21(3):401–5. doi: 10.1038/s41592-024-02168-y (PMC10927542; doi:10.1038/s41592-024-02168-y)
Supplement: Supplementary file 2 — Reporting Summary [file 41592_2024_2168_MOESM2_ESM.pdf]

nature portfolio

Corresponding author(s): Adam P Cribbs

Last updated by author(s): Nov 24, 2023

Reporting Summary

Nature Portfolio wishes to improve the reproducibility of the work that we publish. This form provides structure for consistency and transparency in reporting. For further information on Nature Portfolio policies, see our Editorial Policies and the Editorial Policy Checklist.

Statistics

For all statistical analyses, confirm that the following items are present in the figure legend, table legend, main text, or Methods section.

n/a	Confirmed	
☐	☒	The exact sample size (*n*) for each experimental group/condition, given as a discrete number and unit of measurement
☐	☒	A statement on whether measurements were taken from distinct samples or whether the same sample was measured repeatedly
☒	☐	The statistical test(s) used AND whether they are one- or two-sided<br>*Only common tests should be described solely by name; describe more complex techniques in the Methods section.*
☐	☒	A description of all covariates tested
☒	☐	A description of any assumptions or corrections, such as tests of normality and adjustment for multiple comparisons
☐	☒	A full description of the statistical parameters including central tendency (e.g. means) or other basic estimates (e.g. regression coefficient) AND variation (e.g. standard deviation) or associated estimates of uncertainty (e.g. confidence intervals)
☐	☒	For null hypothesis testing, the test statistic (e.g. F, t, r) with confidence intervals, effect sizes, degrees of freedom and P value noted<br>*Give P values as exact values whenever suitable.*
☒	☐	For Bayesian analysis, information on the choice of priors and Markov chain Monte Carlo settings
☒	☐	For hierarchical and complex designs, identification of the appropriate level for tests and full reporting of outcomes
☒	☐	Estimates of effect sizes (e.g. Cohen's *d*, Pearson's *r*), indicating how they were calculated

Our web collection on statistics for biologists contains articles on many of the points above.

Software and code

Policy information about availability of computer code

Data collection	For PacBio CCS fastq reads were generated using CCS v6.3.0 (https://github.com/PacificBiosciences/ccs). For ONT sequencing, fastq files were generated using guppy v6.4.8 (ONT downloads)
Data analysis	Custom pipelines used within the analysis is available on Github (https://github.com/cribbslab/TallyTriN). External software called by these pipelines include: minimap2 (v2.25); Seurat package (v4.3.0); R/Bioconductor (v4.3.0); pysam (v0.21.0) bustools (v0.42.0); cgatcore (v0.6.15); UMI-tools (v1.1.4); featureCounts (v2.0.3) and hisat2 (v2.2.1). ResimPy (v0.0.1) is available on github (https://github.com/cribbslab/resimpy).

For manuscripts utilizing custom algorithms or software that are central to the research but not yet described in published literature, software must be made available to editors and reviewers. We strongly encourage code deposition in a community repository (e.g. GitHub). See the Nature Portfolio guidelines for submitting code & software for further information.

Data

Policy information about availability of data

All manuscripts must include a data availability statement. This statement should provide the following information, where applicable:

- Accession codes, unique identifiers, or web links for publicly available datasets
- A description of any restrictions on data availability
- For clinical datasets or third party data, please ensure that the statement adheres to our policy

Sequencing data has been deposited to GEO under accession number GSE218899. All analysis was performed using hg38 ensembl 98 version.

Human research participants

Policy information about studies involving human research participants and Sex and Gender in Research.

Reporting on sex and gender	N/A
Population characteristics	N/A
Recruitment	N/A
Ethics oversight	N/A

Note that full information on the approval of the study protocol must also be provided in the manuscript.

Field-specific reporting

Please select the one below that is the best fit for your research. If you are not sure, read the appropriate sections before making your selection.

☒ Life sciences ☐ Behavioural & social sciences ☐ Ecological, evolutionary & environmental sciences

For a reference copy of the document with all sections, see nature.com/documents/nr-reporting-summary-flat.pdf

Life sciences study design

All studies must disclose on these points even when the disclosure is negative.

Sample size

No sample size calculation was performed prior to the study. We chose a minimum of three independent experiments to evaluate our methodology, a decision guided by several key considerations.

Firstly, the principle of replication, which is fundamental to scientific robustness, informed our choice. By conducting the experiment three times independently, we aimed to ensure that our findings were reproducible and not simply due to random variation.

Secondly, our methodology's primary focus is on evaluating the robustness of our assay. Utilising three independent experiments strikes a balance between practical feasibility and obtaining sufficient data to assess the assay's consistency across different runs. This number of experiments was deemed to provide enough data points to enable a meaningful analysis while being feasible in terms of resource allocation and time management.

Additionally, with a sample size of three, we are able to calculate the standard error of the mean (SEM). The SEM is an essential statistical measure for our study, particularly for the UMI/CMI correction, as it provides an estimate of the precision of our sample mean. This is crucial in quantifying the uncertainty associated with the measurements obtained from our assay.

Data exclusions

No data was excluded from the study.

Replication

All of our experiments were replicated in a minimum of three independent experiments unless otherwise stated within the figure legends.

Randomization

A comparative analysis was not undertaken, and therefore blinding was not deemed necessary. However, to provide clarity on the experimental design, we will elaborate on how samples were allocated into experimental groups.

In this study, sample allocation into experimental groups was not performed randomly. This decision was based on the specific objectives and design of our research, which focused on the evaluation of a particular assay or process rather than comparing different groups or conditions. Given the non-comparative nature of the study, the allocation of samples was guided primarily by the need to test the assay under consistent and controlled conditions.

We ensured that the samples were representative of the typical conditions under which the assay would be used, but they were not distributed across different experimental groups for comparative analysis. Therefore, the control of covariates, a critical aspect in comparative

studies to ensure that differences between groups are not due to confounding variables, was not applicable in the context of our study.

Blinding

Blinding was not performed as part of this study. In the our study, the primary aim was to evaluate the performance of a specific assay under consistent conditions, rather than to compare outcomes across different groups or conditions. The focus was on the objective measurement of assay parameters, which are inherently independent of researcher or participant bias. Given the nature of the measurements—likely quantitative and based on predefined, objective criteria—the potential for subjective interpretation or bias was minimal.

Furthermore, the experimental design did not involve variables that could be influenced by the knowledge of the researchers or subjects. For instance, if the study involved chemical or laboratory analyses where outcomes are determined by standardized procedures and equipment, the scope for bias that could be mitigated by blinding is significantly reduced.

Reporting for specific materials, systems and methods

We require information from authors about some types of materials, experimental systems and methods used in many studies. Here, indicate whether each material, system or method listed is relevant to your study. If you are not sure if a list item applies to your research, read the appropriate section before selecting a response.

Materials & experimental systems

n/a	Involved in the study
☒	☐ Antibodies
☐	☒ Eukaryotic cell lines
☒	☐ Palaeontology and archaeology
☒	☐ Animals and other organisms
☒	☐ Clinical data
☒	☐ Dual use research of concern

Methods

n/a	Involved in the study
☒	☐ ChIP-seq
☒	☐ Flow cytometry
☒	☐ MRI-based neuroimaging

Eukaryotic cell lines

Policy information about cell lines and Sex and Gender in Research

Cell line source(s)	Jurkat and RM82 cell lines were purchased from ATCC. 5TGM1 cells were a kind gift from Prof Clair Edwards (University of Oxford).
Authentication	Cell lines were authenticated by STR.
Mycoplasma contamination	Cell lines were mycoplasma tested routinely and all tested negative.
Commonly misidentified lines (See ICLAC register)	No commonly misidentified lines used in this study.
